# GaN Surface Passivation by MoS_2_ Coating

**DOI:** 10.1021/acs.nanolett.4c02259

**Published:** 2024-08-12

**Authors:** Danxuan Chen, Jin Jiang, Thomas F. K. Weatherley, Jean-François Carlin, Mitali Banerjee, Nicolas Grandjean

**Affiliations:** † Laboratory of Advanced Semiconductors for Photonics and Electronics, Institute of Physics, École Polytechnique Fédérale de Lausanne (EPFL), CH-1015 Lausanne, Switzerland; ‡ Laboratory of Quantum Physics, Institute of Physics, École Polytechnique Fédérale de Lausanne (EPFL), CH-1015 Lausanne, Switzerland

**Keywords:** surface passivation, mixed-dimensional van der Waals heterostructures, III-nitride semiconductors, two-dimensional transition metal dichalcogenides, cathodoluminescence

## Abstract

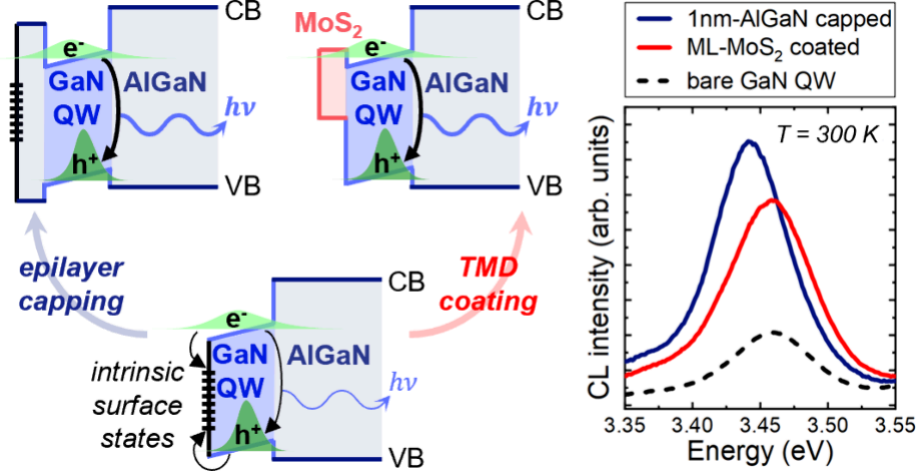

In this study, we investigate the impact of two-dimensional MoS_2_ coating on the optical properties of surface GaN/AlGaN quantum wells (QWs). A strong enhancement in GaN QW light emission is observed with monolayer-MoS_2_ coating, yielding luminescence intensity comparable to that from a QW capped by an AlGaN barrier. Our results demonstrate that MoS_2_, despite its quite different nature from III-nitride semiconductors, acts as an effective barrier for surface GaN QWs and suppresses spatially localized intrinsic surface states. This finding provides novel pathways for efficient III-nitride surface passivation.

Since the breakthroughs of blue light-emitting diodes (LEDs) in the 1990s,^1^ III-nitrides have emerged as a major semiconductor family. On the other hand, the isolation of graphene in 2004^2^ marked the inception of a new era in solid-state physics. Among various two-dimensional (2D) materials, transition-metal dichalcogenides (TMDs), such as MoS_2_, exhibit a sizable bandgap,^3^ strong light–matter coupling,^4^ and robust excitonic features,^5^ making them highly desirable for optoelectronic applications.

Mixed-dimensional van der Waals (vdW) heterostructures combining TMDs with III-nitrides have already been proposed for a diverse range of applications including LEDs,^6^ water splitting,^7^ and photodetection.^8^ Conventional semiconductors possess surface states (SSs) that act as nonradiative recombination centers (NRCs).^9^ However, their bonding with 2D materials could change the surface electronic structure;^10^ charge transfer can occur by tunneling and/or hopping,^11^ which could passivate SSs in III-nitride semiconductors.^12,13^

In this study, we deposit MoS_2_ on a series of surface polar GaN/AlGaN quantum wells (QWs) with varying AlGaN top barrier thickness *d* (*d* = 0–15 nm). Cathodoluminescence (CL) measurements on the uncapped QW (*d* = 0 nm) show appreciable emission at room temperature (RT). Upon coating the surface with a single MoS_2_ monolayer (ML), the CL intensity is enhanced; while for all samples with *d* > 0 nm, the QW emission decreases with increasing MoS_2_ thickness, consistent with MoS_2_ absorption.^14,15^ The possible origins of the MoS_2_-enhanced emission for *d* = 0 nm are discussed, followed by a comparison between the emissions of the QWs coated by ML-MoS_2_ and 1 nm AlGaN barrier. This highlights MoS_2_ as an efficient surface barrier in the hybrid MoS_2_/GaN/AlGaN QW system.

III-nitride samples are grown by metalorganic vapor phase epitaxy (Supporting Information (SI), Sec. 1). The structure consists of a polar surface single GaN/Al_0.1_Ga_0.9_N QW (Figure 1a). A 500 nm thick AlGaN spacer is inserted beneath the QW to prevent parasitic luminescence from the GaN buffer (SI, Sec. 2). The RT CL spectra of the samples display emission peaks at ∼3.44 and ∼3.63 eV, attributed to the surface GaN QWs and AlGaN spacers, respectively (Figure 1b). The first important observation is that, in contrast to near-surface GaAs QWs,^16^ all GaN QWs exhibit an appreciable light emission at RT, even in the absence of a surface barrier (SI, Sec. 3). This confirms the lower impact of nonradiative surface recombination in III-nitrides compared to other III–V semiconductors.^17^

**Figure 1:**
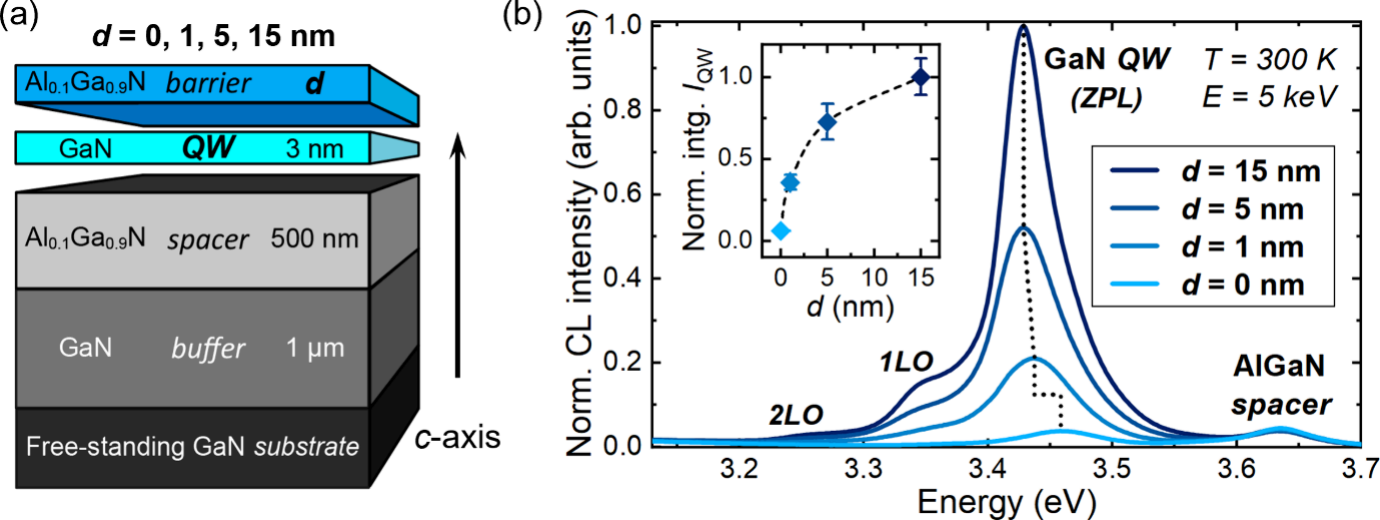
(a) Sample structure of surface QWs and (b) corresponding CL spectra acquired at 300 K under an electron beam energy of 5 keV. The peaks corresponding to the AlGaN spacer emission, as well as the zero-phonon line (ZPL) and the longitudinal optical (LO) phonon replicas of the GaN QW emission, are identified. In the inset, the integrated QW intensity (*I*_QW_), including only the ZPL, is plotted as a function of surface barrier thickness (*d*). Some intensity error bars are not visible in the plot, as they are smaller than the size of the diamond symbol used.

Now we will delve into the characteristics of the QW optical properties. As the thickness of the surface barriers is much smaller than the carrier diffusion length in Al_0.1_Ga_0.9_N,^18^ one can assume that carriers generated in the surface barrier either nonradiatively recombine at the surface or diffuse toward the QW. Hence, the AlGaN emission in Figure 1b originates from the spacer. The depth of the interaction volume of the 5 keV electron beam in these samples is more than 100 nm (SI, Sec. 2), which implies that the position of the QW with respect to the surface, i.e., *d*, does not influence carrier injection into both the GaN QW and AlGaN spacer, as testified by the comparable AlGaN CL intensity in all the samples (SI, Sec. 3). In contrast to QWs with a top AlGaN barrier, the peak of the uncapped well (*d* = 0 nm) undergoes a notable blueshift of ∼30 meV. This can be explained by a stronger carrier quantum confinement imposed by the free surface (SI, Sec. 3) and a reduction in the quantum-confined Stark effect (QCSE) in the QW. QCSE is huge in III-nitride heterostructures grown along the *c*-axis, owing to the large polarization mismatch at heterointerfaces.^19^ However, for *d* = 0 nm, the built-in field is weaker due to the absence of the GaN/AlGaN interface, resulting in a blueshift of the QW emission.^20^ Interestingly, under the same injection conditions, the integrated QW intensity (see SI, Sec. 3 for calculation details) in Figure 1b exhibits a nonlinear increase with increasing *d*. For a *c*-plane III-nitride surface, a high density of deep levels can act as effective NRCs.^21,22^ Therefore, the nonlinear increase in QW emission can be attributed to the increasing distance of the QW from the surface, which will be discussed later. This notable increase also demonstrates the significant impact of the SSs, despite the low surface recombination velocity usually ascribed to III-nitrides.^17^ This highlights the importance of surface passivation in this materials system, particularly for photonic devices with a high surface-to-volume ratio, such as micro-LEDs.^23,24^

Mechanically exfoliated MoS_2_ flakes were prepared on a SiO_2_/Si substrate, where the contrast in an optical microscope is highly sensitive to MoS_2_ thickness due to light interference^25^ (Figures 2a,d). After precise characterization of the layer thickness by atomic force microscopy and Raman spectroscopy (SI, Sec. 4), the selected flakes were deposited on the surface GaN QWs. Hyperspectral CL maps were acquired on the MoS_2_ flake regions. All intensity maps were normalized by the average intensity of the background (SI, Sec. 5).

**Figure 2:**
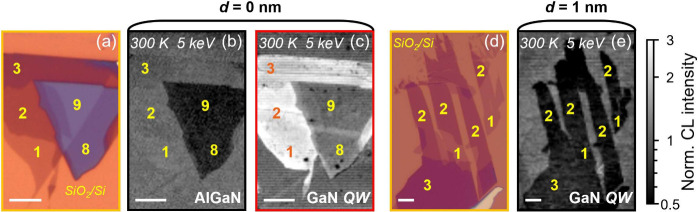
(a, d) Optical micrographs of the selected MoS_2_ flakes on a SiO_2_/Si substrate. Normalized integrated CL intensity maps of the (b) AlGaN and (c) GaN QW emissions from the uncapped GaN QW (*d* = 0 nm), as well as (e) the GaN QW emission from the sample with *d* = 1 nm, acquired with an electron beam energy of 5 keV at 300 K. For each map, the normalization was performed using the average intensity in the region without MoS_2_. All CL maps are plotted on a logarithmic intensity scale ranging from 0.5 to 3. The numbers in yellow/orange indicate the number of MoS_2_ MLs in the corresponding region. Scale bars correspond to a length of 5 μm.

The intensity maps show three different contrasts (Figure 2b,c,e). The AlGaN spacer map, extracted from the uncapped GaN QW (*d* = 0 nm), is straightforward to interpret (Figure 2b). Areas covered by 1–3 MoS_2_ MLs display an intensity close to the background, while those covered by 8 and 9 MLs appear significantly darker. Clearly, there is a gradual decrease in CL intensity as MoS_2_ thickness increases due to absorption, which scales proportionally with the number of MLs.^15^ For the QW with *d* = 1 nm (Figure 2e), the CL intensity also reduces with the presence of MoS_2_. However, the quenching in QW emission is notably stronger compared to that of the spacer (Figure 2b). This difference is not aligned with the similar spectral absorptance of MoS_2_ in the spectral range of GaN QW and AlGaN spacer emissions (where ∼10% of the incident light is absorbed by ML-MoS_2_,^14^ detailed in SI, Sec. 6). The origin of this “enhanced absorption” will be the focus of future study. At the same time, the significant quenching of surface QW emission by MoS_2_ coating highlights the peculiarity of the GaN QW emission for *d* = 0 nm (Figure 2c): the regions covered by 1–3 MLs of MoS_2_ exhibit a high intensity, while the regions covered by 8 and 9 MLs appear darker but comparable to the background, despite the strong absorption depicted in other maps (Figure 2b,e). This CL intensity behavior likely results from a combination of QW emission enhancement due to the deposition of MoS_2_ and thickness-dependent MoS_2_ absorption of the QW emission. Since the enhancement disappears when slightly moving the QW away from the surface, i.e., *d* = 1 nm, it is likely that the enhanced GaN emission is associated with the MoS_2_/III-nitride vdW interface.

To confirm this hypothesis, we performed CL experiments on a GaN epilayer coated by MoS_2_ (SI, Sec. 7). The CL map also exhibits an increase in GaN emission in the presence of MoS_2_, albeit weaker compared to the case of the uncapped GaN QW. This is consistent with a surface effect: in a GaN epilayer, CL emission comes from both the surface and bulk regions.

To gain more quantitative insights, we segmented the CL map of the uncapped QW (*d* = 0 nm) into regions with varying MoS_2_ thicknesses (SI, Sec. 5). The average CL spectra extracted from regions coated by MoS_2_ of different thicknesses are compared in Figure 3a, which reveals clear opposite changes in the GaN QW and AlGaN spacer peak intensities when transitioning from uncoated (0*L*) to MoS_2_-coated regions (>0*L*). The histogram of integrated intensities in each region is fitted with a normal distribution (SI, Sec. 5) and the resulting mean value is plotted as a function of MoS_2_ thickness (Figure 3b,c). Let us consider first the AlGaN spacer emission (Figure 3b). Except for the slight increase in intensity from 0*L* to 1*L*, the AlGaN intensity decreases monotonically with increasing MoS_2_ thickness, as expected from MoS_2_ absorption. Noticeably, having a few MLs of MoS_2_ has a negligible impact on carrier injection (SI, Sec. 2), primarily due to their limited interaction with the electron beam.^26^ To model the AlGaN intensity decrease, we consider that the intermonolayer coupling in MoS_2_ does not strongly influence the absorption. Therefore, the intensity can be fitted with a power function: *I*(*n*) = *I*_0_·(1 – *a*)^*n*^, where *I*_0_ = *I*(*n* = 0), *n* is the number of MoS_2_ MLs, and *a* is the absorptance in each ML. We deduce *a* ≈ (8 ± 3)%, which agrees well with the absorptance of ML-MoS_2_ at the peak energy of AlGaN emission^14^ (SI, Sec. 6). The small increase in AlGaN intensity between 0*L* and 1*L* will be discussed later. Similarly, Figure 3c shows the plot of the GaN QW CL intensity as a function of MoS_2_ thickness. Fitting the data with the same absorption model reproduces the overall trend, with *a* ≈ (12 ± 3)%, which is well in line with the MoS_2_ absorptance at the QW peak energy^14^ (SI, Sec. 6). Interestingly, the fit fails to capture the data at *n* = 0, instead predicting an intensity ∼3.2 times higher than the measured value. This indicates that the deposition of the first ML-MoS_2_ results in a strong increase in the emission of the uncapped QW.

**Figure 3:**
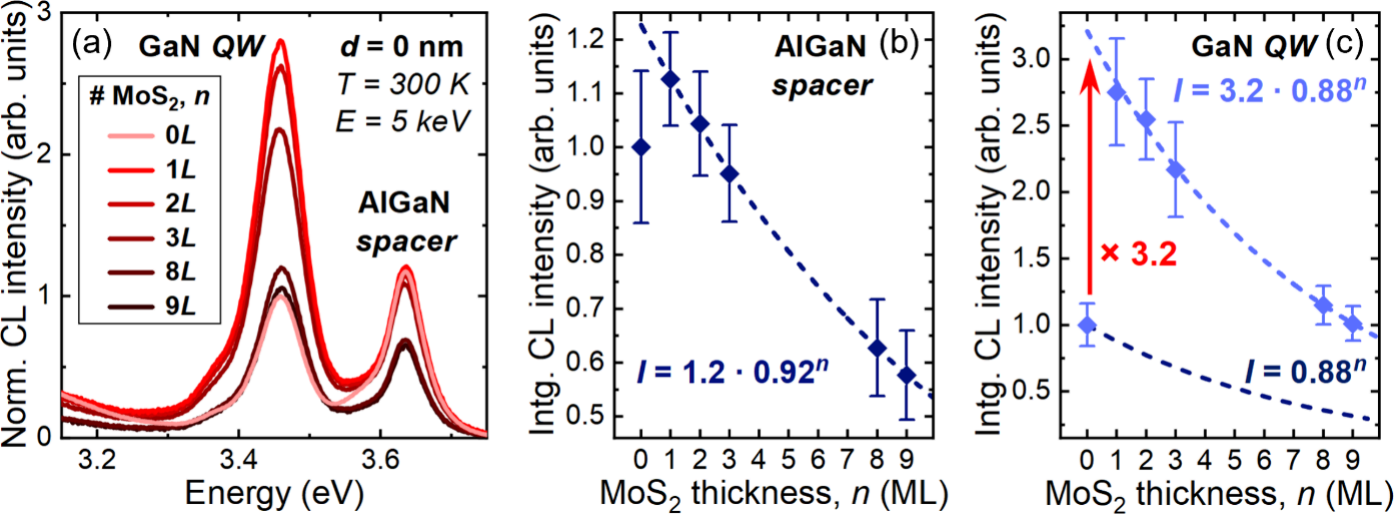
(a) Average RT CL spectra collected from the uncapped GaN QW (*d* = 0 nm) in areas with varying MoS_2_ thicknesses, represented by the number of MLs (*n*). All spectra are normalized to the peak intensity of the GaN QW emission in the region without MoS_2_ (0*L*). Normalized integrated CL intensity as a function of MoS_2_ thickness for (b) the AlGaN spacer emission and (c) the GaN QW emission. The dashed lines represent the fits assuming the same absorption is occurring in each ML-MoS_2_, with the corresponding expression next to them.

To understand this effect, we should consider various mechanisms, such as changes in carrier injection, light extraction, and recombination rate. As mentioned earlier, the interaction between the electron beam and ML-MoS_2_ is negligible (SI, Sec. 2) and, even so, it should decrease the number of injected carriers in the QW. On the other hand, ML-MoS_2_ could modify the band bending^8,27,28^ and thereby enhance carrier transfer from the AlGaN spacer to the QW. However, the AlGaN spacer intensity increases upon 1 ML-MoS_2_ deposition (Figure 3b), which rules out this hypothesis.

Another factor to consider is an increase of the light extraction due to Fabry–Perot cavity effect, as for 2D materials deposited on SiO_2_/Si substrates.^29,30^ However, at ∼360 nm, the QW emission wavelength, the refractive indices of MoS_2_, GaN, and Al_0.1_Ga_0.9_N are comparable (SI, Sec. 8). Also, a 1 nm change in the QW position should not impact photon extraction.

A modification in the radiative recombination rate could also be at play: the deposition of MoS_2_ might alter the band bending of the surface region,^8,27,28^ which in turn might reduce the internal electric field in the surface QW, i.e., the QCSE. However, no significant CL peak energy change is observed upon MoS_2_ deposition (SI, Sec. 8). This is also consistent with the rather high injected carrier density in the QW (∼10^12^ cm^–2^, estimated in SI, Sec. 2), which induces a partial screening of the built-in field (see QW emission under various injection conditions in SI, Sec. 3). Notice that an emission intensity increase was also observed for bulk GaN epilayer upon MoS_2_ coating (SI, Sec. 7).

Another explanation for the increased intensity is a protection of the surface: carriers in the surface QW are very sensitive to surface contamination upon electron beam irradiation.^31^ In fact, the *c*-plane III-nitride surface is polar and may trap residues of hydrocarbons used during the MoS_2_ transfer (SI, Sec. 1). These residues could act as a carbon source for surface contamination,^32^ leading to a reduction in surface emission in the uncoated area, and consequently, a relative enhancement in the region covered by MoS_2_. To check this hypothesis, we conducted a subsequent scan on the QW with *d* = 0 nm (Figure 4a,b). Unlike the AlGaN spacer CL peak, the surface QW emission from the uncoated region (0*L*) is significantly reduced after the first measurement (Figure 4c), which may support the presence of surface contamination. However, the increase in QW peak intensity induced by MoS_2_ coating remains similar for the two scans. If MoS_2_ were protecting the surface QW from any contamination, each scan should further reduce the emission in the uncoated region and have no impact on the “protected” region. Consequently, the difference between the two areas should be more pronounced for the second scan, which contradicts our observations. Hence, the MoS_2_-induced change in the CL intensity of the QW with *d* = 0 nm cannot be attributed to an electron-beam-induced contamination. Actually, the slight reduction of the CL intensity enhancement observed in the second scan (Figure 4b) is likely related to the degradation of MoS_2_ under electron irradiation^33−35^ (SI, Sec. 9). This indicates that the 3.2-factor enhancement in CL emission from the surface QW coated by 1 ML-MoS_2_ is underestimated.

**Figure 4:**
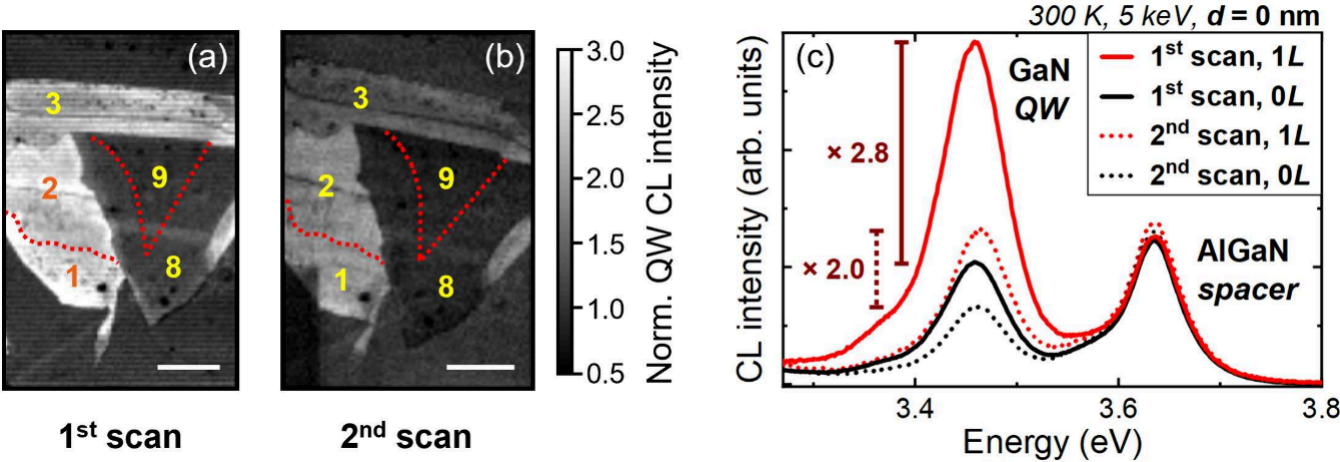
RT integrated GaN QW CL intensity maps of (a) the 1st scan and (b) the 2nd scan, where the numbers in yellow/orange indicate the number of MoS_2_ MLs in the corresponding region. The red dotted lines are drawn to highlight unclear boundaries between different regions, based on the MoS_2_ thickness-dependent color contrast shown in Figure 2a. Both maps are normalized by the respective average intensity in the region without MoS_2_, and are plotted using a linear intensity scale ranging from 0.5 to 3. Scale bars correspond to a length of 5 μm. The dark rectangular area in the left lower corner of the 2nd map is a result of a small-scale measurement conducted prior to this scan. (c) Average CL spectra of the background emission (0*L*) and the emission from the region covered by ML-MoS_2_ (1*L*), extracted from the two scans. The dark rectangular area in the 2nd map is excluded from the estimation of the average spectra.

Eventually, the surface emission increase when the sample is coated by MoS_2_ could be ascribed to a reduction of SSs caused by charge transfer between the two materials. Specifically, since no special treatments, such as oxidation or nitridation, were performed on the GaN surface, and MoS_2_ transfer was carried out in a dry environment (SI, Sec. 1), the MoS_2_/GaN interface is expected to exhibit a type-II band alignment.^7^ Such alignment can result in the passivation of SSs in GaN by charges transferred from the MoS_2_ coating (detailed in SI, Sec. 7). This phenomenon could also account for the slight increase in the AlGaN spacer emission (Figure 3b).

In Figure 5a, we plotted the *d*-dependent QW intensity computed from the average spectra extracted from regions uncoated and coated by 1, 2, and 3 MLs of MoS_2_ (all the CL spectra are presented in SI, Sec. 5). The CL intensity is normalized to the QW with *d* = 15 nm to correct it from MoS_2_ absorption. All the data aligned well except for the uncapped QW (*d* = 0 nm) without MoS_2_ coating (0*L*). In this QW, the carriers are subjected to a high density of intrinsic surface states (ISSs), which are formed due to the termination of the crystal lattice at the surface.^36^ Once the surface is coated by MoS_2_, the empty ISSs become occupied through vdW bonding and/or charge transfer, which effectively reduces their nonradiative recombination activity. However, the further increase in CL intensity with *d*, while the QWs are coated by MoS_2_, suggests another nonradiative recombination mechanism. To understand this, we consider that the recombination of carriers in the QWs through NRCs requires a spatial overlap of electron and hole wave functions with the corresponding defects. Since both electron and hole wave function penetrations into the AlGaN barriers are limited to ∼1 nm, and holes in the well are repelled from the surface by the residual built-in field, tunneling of both electrons and holes toward the ISSs is unlikely with a 1 nm thick barrier (SI, Sec. 10). Thus, the intensity increase observed from 1 to 15 nm cannot be attributed to carrier tunneling. Alternatively, divacancies are one of the main NRCs in (Al)GaN materials,^37^ with nitrogen vacancy known to segregate toward the surface.^38,39^ Recent results show that the concentration of vacancy-related defects in GaN gradually decreases from the surface to the bulk.^40^ Since divacancies are imperfections in the lattice, their associated energy levels are called “extrinsic surface states (ESSs)”.^41^ Therefore, we propose that the QW intensity variation observed from *d* = 1 to 15 nm is linked to the spatial distribution of ESSs probed by QWs located at different depths from the surface. We model this variation by considering an exponential spatial distribution of the ESSs (SI, Sec. 10):
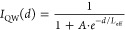
1where *L*_eff_ is a phenomenological parameter used to account for the spatial spreading of ESSs in the (Al)GaN near-surface region and *A* is related to the nonradiative surface recombination rate. This model enables not only to fit well the data from *d* = 1–15 nm, but also aligns well with the data at *d* = 0 nm, except for the QW without MoS_2_ coating (0*L*) (Figure 5a). As expected, when *d* = 0 nm and without any MoS_2_ coating, the model fails to predict the QW intensity due to the presence of ISSs (Figure 5b). Once coated by MoS_2_, ISSs are passivated and the surface QW emission is then mainly limited by ESSs present in the well (Figure 5b). This explains why, with MoS_2_ coating, all the points at *d* = 0 nm are well accounted for by the model (Figure 5a). Therefore, we propose that the deposition of MoS_2_ mainly leads to a strong reduction in ISSs present on the III-nitride surface, which results in a strong enhancement in the luminescence intensity emitted from the surface (i.e., the uncapped GaN QW). Notice that MoS_2_ passivation of ISSs also explains the slight increase in emission from the bulk region (see, e.g., the AlGaN spacer).

**Figure 5:**
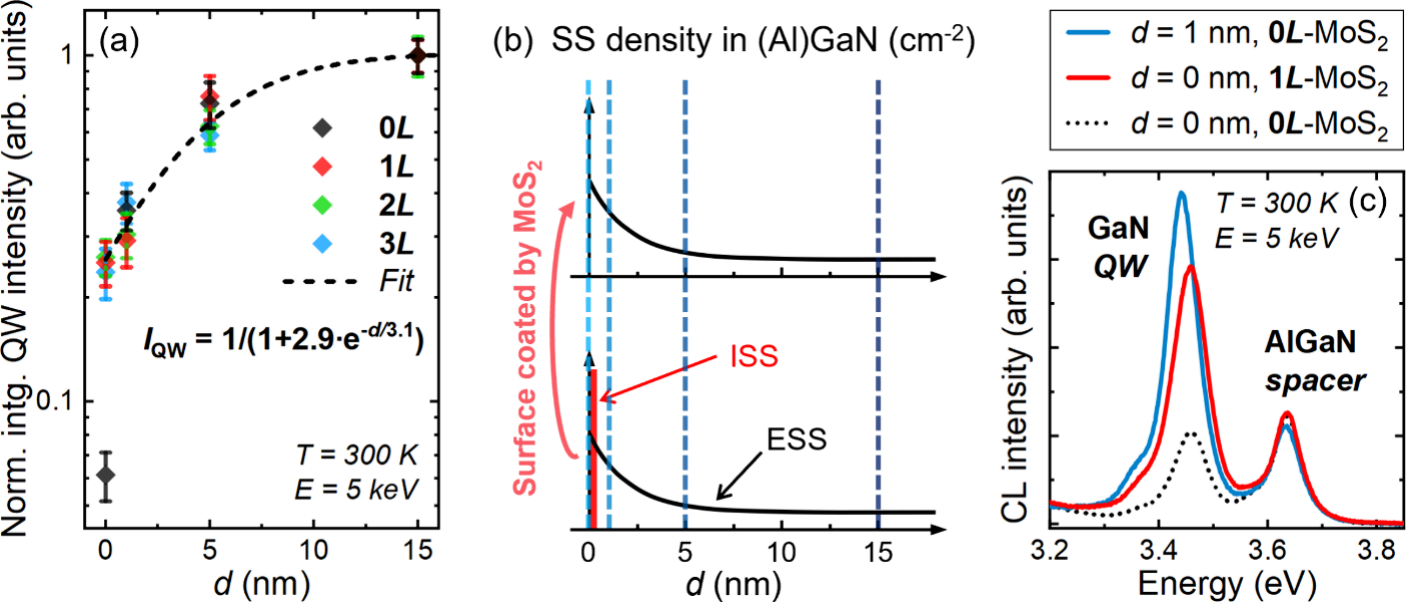
(a) *d*-dependent integrated QW CL intensity, extracted from regions uncoated and coated by MoS_2_ of 1–3 MLs, excited by a 5 keV electron beam at 300 K. All the data, except for the point at *d* = 0 nm for regions without MoS_2_ (0*L*), were fitted by an exponential function based on the spatial distribution of ESSs in III-nitrides, with the corresponding expression shown in the plot. (b) Schematic representation of SS density in (Al)GaN as a function of depth from the surface (*d*). The diagram accounts for both ISSs localized at the surface (depicted by the red line) and ESSs, whose concentration exponentially decreases from the surface into the bulk (illustrated by the black curves). The lower plot shows a bare (Al)GaN surface, while the upper plot represents a surface coated by 2D MoS_2_. The four blue dashed lines indicate the position of the surface/upper interface of the surface GaN QWs with *d* = 0, 1, 5, and 15 nm. (c) Comparison of the average RT CL spectra of the uncapped GaN QW (*d* = 0 nm, 0*L*), as well as QWs coated by ML-MoS_2_ (*d* = 0 nm, 1*L*) or capped by 1 nm Al_0.1_Ga_0.9_N (*d* = 1 nm, 0*L*).

To assess the efficiency of single ML-MoS_2_ as a GaN QW “barrier”, we compared the average CL spectra of the ML-MoS_2_-coated GaN QW (*d* = 0 nm, 1*L*) and the 1 nm-Al_0.1_Ga_0.9_N capped GaN QW (*d* = 1 nm, 0*L*). As depicted in Figure 5c, the GaN QW emission from the hybrid MoS_2_/GaN/AlGaN QW is comparable to that measured on the epitaxially grown AlGaN/GaN/AlGaN QW under identical injection conditions. This indicates that by manipulating the TMD/III-nitride interaction at the vdW interface, one could potentially design efficient vdW capping for III-nitride surfaces. Moreover, the choice of TMD materials is no longer restricted by lattice matching imposed by epitaxial growth; even vdW heterostructures containing various 2D materials could be used as a capping layer with specially designed functionalities. For real-world applications, large-scale 2D material coatings grown via chemical vapor deposition with long-term stability, ensured by optimized growth conditions and structural engineering such as encapsulation, hold promise as passivation layers for III-nitride optoelectronic devices.

In summary, we investigated the optical properties of a series of surface GaN/AlGaN QWs with varying nanometer-scale surface barrier thickness, *d* = 0 to 15 nm. Thanks to a reduced surface recombination rate, high CL intensity was observed, even from the uncapped QW (*d* = 0 nm). However, the QW intensity increases nonlinearly with increasing *d*, highlighting the non-negligible impact of deep traps existing near the *c*-plane III-nitride surface region. Using these surface GaN QWs as a probe light source, we deposited MoS_2_ flakes of a few MLs. The presence of MoS_2_ strongly enhances the light emission from the uncapped QW. Based on our results, we propose that the primary role of MoS_2_ is to passivate intrinsic states at the GaN surface. This proves that the limiting factor for surface III-nitride emission lies in the formation of NRCs due to the termination of the crystal lattice at the surface. This detrimental effect can be mitigated by coating the surface with vdW layers. Importantly, NRCs due to lattice imperfections, like vacancies, are still present at the near surface, which suggests that careful growth optimization is necessary. Overall, our finding demonstrates efficient III-nitride surface passivation by 2D TMD coating, which could be applied to develop micro- and nanoscale optoelectronic devices featuring a high surface-to-volume ratio, such as micro-LEDs.

## Supporting Information Available

1. Experimental methods; 2. Carrier injection in CL; 3. Optical properties of surface GaN QWs; 4. Thickness determination of MoS_2_; 5. CL data processing; 6. Spectral absorptance of ML-MoS_2_; 7. MoS_2_ on the bulk GaN epilayer; 8. Possible surface passivation mechanisms; 9. Degradation of MoS_2_ under electron beam irradiation; 10. Model for the *d*-dependent QW intensity (PDF)
