# Supplementary material for: GaN Surface Passivation by MoS2 Coating
Source: Nano Lett. 2024 Aug 12;24(33):10124–30. doi: 10.1021/acs.nanolett.4c02259 (PMC11342355; doi:10.1021/acs.nanolett.4c02259)
Supplement: Supplementary file 1 — nl4c02259_si_001.pdf [file nl4c02259_si_001.pdf]

Supporting Information:
GaN Surface Passivation by MoS_2 Coating

Danxuan Chen,[*,†] Jin Jiang,[‡] Thomas F. K. Weatherley,[†] Jean-François Carlin,[†] Mitali Banerjee,[‡] and Nicolas Grandjean[†]

†*Laboratory of Advanced Semiconductors for Photonics and Electronics (LASPE)*

‡*Laboratory of Quantum Physics (LQP)*

École Polytechnique Fédérale de Lausanne (EPFL), CH-1015 Lausanne, Switzerland

E-mail: danxuan.chen@epfl.ch

Phone: +41 (0)21 693 45 33

1. Experimental methods

Sample growth. The samples used in this study were grown by metalorganic vapor phase epitaxy in a horizontal Aixtron 200/4 RF-S reactor on commercial *c*-plane free-standing GaN substrates with very low dislocation density, typically $\sim 10^6$ cm^{-2}. The growth process can be divided into two parts. First, a GaN buffer and an $\sim$ 500 nm $Al_{0.1}Ga_{0.9}N$ spacer are deposited at a growth rate of ~ 2 μm/h and a temperature of 1000 °C. Trimethylgallium and trimethylaluminum are used as precursors, and H_2 is used as carrier gas. Then, the growth rate is lowered to 60 nm/h at a temperature of 800 °C for the growth of the single quantum well (QW) region, which includes a 5 nm $Al_{0.1}Ga_{0.9}N$ barrier, the 3 nm GaN QW layer, and the $Al_{0.1}Ga_{0.9}N$ surface barrier. This low-temperature (LT) growth is intended to mitigate large-scale Al content fluctuations within the barriers, thereby minimizing the inhomogeneous broadening of the QW emission.[1] For the LT growth, the metalorganic source

for gallium is changed to triethylgallium, and the carrier gas is switched to N_2. The entire structure is grown without any intentional doping.

Fabrication of MoS_2-on-(Al)GaN heterostructures. The MoS_2 flakes were obtained through the well-known "scotch-tape" mechanical exfoliation method[2] and deposited on a 10-minute oxygen-plasma-etched SiO_2/Si substrate. The precise thickness of the selected MoS_2 flakes was determined by optical microscopy, atomic force microscopy (AFM) and Raman spectroscopy (Sec. 4). Following the characterization, the selected flakes were picked up and transferred onto the cleaned surface of the (Al)GaN samples (QWs and a bulk GaN epilayer) using a dry transfer technique.[3,4] Initially, a high-quality uniform stack of poly(bisphenol A carbonate)/polydimethylsiloxane (PDMS) was prepared on a glass slide. This stack was then mounted on a homemade transfer stage to pick up MoS_2 at 70 °C. Subsequently, the MoS_2 flake was transferred to the surface of the (Al)GaN sample at 150 °C. Unlike the SiO_2/Si substrate, no plasma treatment, which could damage the (Al)GaN surface, was performed on the samples. Finally, the entire sample was immersed in chloroform to clean its surface.

Cathodoluminescence spectroscopy. Cathodoluminescence (CL) imaging was conducted using an advanced scanning electron microscope system (Attolight Rosa 4634) with an acceleration voltage of 5 kV. A Cassegrain reflective objective was employed to collect the emitted light, which was subsequently directed to a spectrometer equipped with a 600 lines per mm grating with a blaze wavelength of 300 nm. The dispersed light was then captured by a cooled charge-coupled device camera, enabling the recording of a full intensity-energy spectrum at each pixel, i.e., hyperspectral imaging.

2. Carrier injection in CL

The interaction volume of a 5 keV electron beam at 300 K in bulk $Al_{0.1}Ga_{0.9}N$ is determined through Monte Carlo simulation (*CASINO*),[5] as depicted in Fig. S1a. For this simulation, an electron beam containing 1×10^6 electrons was used, with a spot size of 25 nm,

and an accelerating voltage of 5 kV. The density of $Al_{0.1}Ga_{0.9}N$ is $\rho = 5.86$ g/cm^3. This can be deemed representative of all the QW samples examined in this study, as the thickness of the GaN QW is negligible compared to the overall interaction volume, and a 10% variation in Al content leads to less than 5% change in mass density. Based on the simulation, the normalized energy absorbed in the sample is plotted as a function of depth from the surface (Fig. S1b), which indicates that most of the beam energy is absorbed within an $\sim$ 150 nm region from the surface. Considering that the minority carrier diffusion length in GaN and $Al_{0.1}Ga_{0.9}N$ is typically limited to 100 nm at room temperature (RT),[6,7] the 500 nm thick $Al_{0.1}Ga_{0.9}N$ spacer serves as an effective barrier, which prevents beam-generated carriers from reaching the GaN buffer. Consequently, the observed GaN emission in all the samples is attributed solely to the GaN QWs.

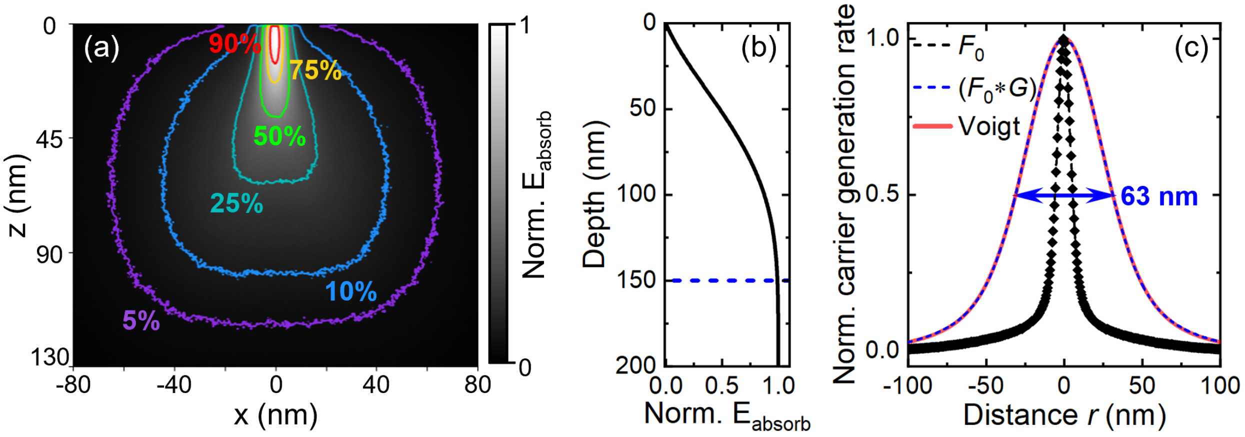

Figure S1: (a) Cross-section view of absorbed energy resulting from the Monte Carlo simulation of a 5 keV electron beam interacting with bulk $Al_{0.1}Ga_{0.9}N$ at 300 K. (b) Depth-dependent energy deposition in the sample, normalized by the total absorbed energy. (c) Lateral carrier generation rate distribution, $F(r)$, derived by convolving the simulated carrier generation rate distribution, $F_0(r)$, with a Gaussian function, $G(r)$, characterized by a standard deviation $\sigma = 22$ nm, accounting for the broadening due to carrier thermalization at 300 K.[8] The profile is fitted by a Voigt function with a full width at half maximum (FWHM) of $\sim$ 63 nm.

To estimate the carrier density in the QWs (n_{QW}), we first compute the lateral carrier generation rate distribution $F(r)$, where r represents the lateral distance to the beam center using cylindrical coordinates. This is achieved by convolving the carrier generation rate

distribution $F_0(r)$, deduced from the simulation, with a Gaussian distribution $G(r)$. Specifically, $F_0(r)$ is obtained by summing the deposited energy over the z direction, assuming that all carriers relax to the QW. $G(r)$ is characterized by a standard deviation $\sigma = 22$ nm, which accounts for the broadening caused by carrier thermalization at 300 K.[8] The resulting profile is normalized by its peak value at $r = 0$ and fitted by a Voigt function, yielding a FWHM of ~ 63 nm (Fig. S1c). It is important to note that our estimation does not consider the lateral carrier diffusion occurring in the barrier before carriers completely relax to the QW. As a result, the calculated FWHM underestimates the actual broadening of the carrier generation rate distribution in the QW. Meanwhile, the total generation rate, G_{tot} (s^{-1}), of carriers in CL can be estimated using the well-known equation:[9]

$$G_{\text{tot}} = \frac{I_{\text{p}}}{q} \cdot \frac{E_{\text{dep}}}{3E_{\text{g}}} , \tag{1}$$

where I_{p} is the electron beam probe current, q is the charge of an electron, E_{dep} is the average energy deposited per electron in the sample, and E_{g} is the bandgap of the sample. E_{dep} is calculated as the difference between the beam energy (E_{beam}) and the energy lost through backscattered electrons (E_{BSE}). In our case, $I_{\text{p}} = 221$ to 233 pA, measured using a Faraday cup attached to the sample holder, $E_{\text{g}} = 3.64$ eV for $Al_{0.1}Ga_{0.9}N$ at 300 K,[10] $E_{\text{beam}} = 5$ keV, and $E_{\text{BSE}} \approx 1.14$ keV computed via Monte Carlo simulation. Assuming that all generated carriers relax to the QW, the carrier generation rate in the QW, G_{QW}, is around $5 \cdot 10^{11}$ s^{-1}. Under the assumptions of carrier lifetime being independent of carrier density and no carrier diffusion in the QW, the maximal carrier density in the QW at $r = 0$ can be calculated as:

$$n_{\text{QW}}^{\text{max}} = \frac{G_{\text{QW}} \cdot \tau}{2\pi \int_0^{\infty} F(r) r \mathrm{d}r} , \tag{2}$$

with τ the carrier lifetime, which is approximately 1 ns for a single $GaN/Al_{0.1}Ga_{0.9}N$ QW at RT under low injection condition.[11] Considering the higher injection in our CL measurements, this value should be regarded as an upper limit. The estimated maximal carrier

density in the QWs is approximately $8 \cdot 10^{12}$ cm^{-2}. It should be noted that this value may be overestimated, considering the broader actual carrier distribution and the shorter actual carrier lifetime, as discussed previously. Additionally, our assumption that all carriers relax to the QW might not hold true in practice. As a result, the average carrier density in the QWs is expected to be on the order of 10^{12} cm^{-2}.

In order to study the impact of MoS_2 flakes on the injection of carriers into the GaN QWs, we simulate the interaction of the primary electron beam with MoS_2 of a few monolayers (MLs) using *CASINO*. To simplify the situation, we consider again bulk $Al_{0.1}Ga_{0.9}N$ as representative for all the samples in the study and simulate the percentage of energy absorbed in the two-dimensional (2D) MoS_2 as a function of MoS_2 thickness (Fig. S2). The ML-MoS_2 thickness is set to 0.7 nm, and the density of MoS_2 is $\rho = 5.06$ g/cm^3.[12] The simulated results reveal a linear dependence of the energy absorbed in MoS_2 on the MoS_2 thickness, $\sim 0.4\%$ per ML (Fig. S2c). Notably, the percentage of absorbed energy we found here is two orders of magnitude larger than the percentage of scattering events in 2D MoS_2 reported by M. Negri *et al.*[12] Although the substrates differ, given that most of the primary beam's energy is lost through scattering in the surface region, it is reasonable to find a much stronger contribution

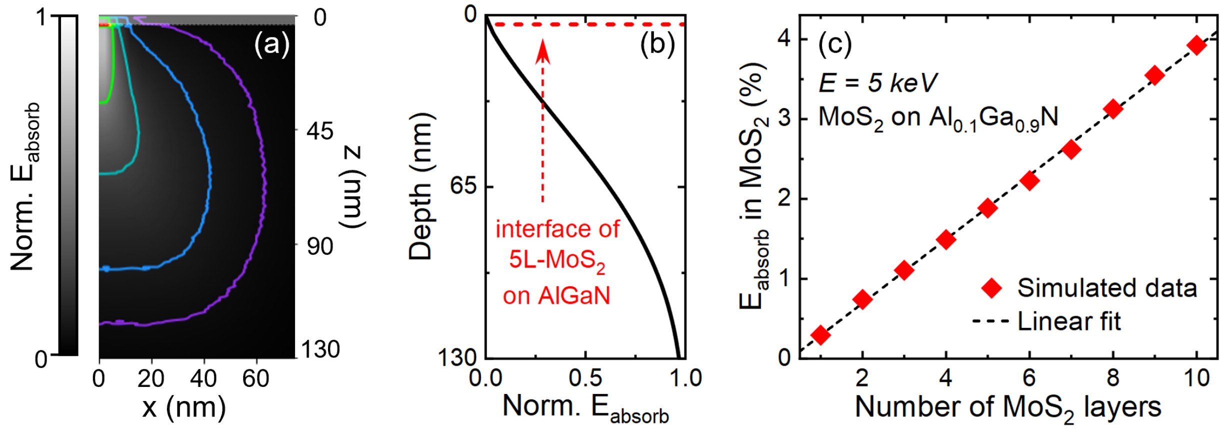

Figure S2: (a) RT cross-section view of absorbed energy of a 5 keV electron beam interacting with bulk $Al_{0.1}Ga_{0.9}N$ coated by MoS_2 of 5 MLs. The shaded region represents the MoS_2 flake. (b) The associated depth-dependent energy deposition in the sample, normalized by the total absorbed energy. (c) Percentage of absorbed beam energy in the MoS_2 layer as a function of MoS_2 thickness.

of interaction in the 2D layer when considering the absorbed energy instead of scattering events. However, this discrepancy does not affect our absorption fit in Figs. 3b and 3c of the main text, as we found $\sim 10\%$ change in CL intensity induced by each ML-MoS_2. This value is significantly larger than the $\sim 0.4\%$ beam energy absorbed by each ML. Therefore, we can conclude that the monotonic decrease in (Al)GaN CL intensity with increasing MoS_2 thickness is mainly due to MoS_2 absorption of the CL emission, rather than electron beam energy absorbed in the 2D layer.

3. Optical properties of surface GaN QWs

Since the QW emission varies significantly with the surface barrier thickness, d, CL spectra of the surface GaN QWs are presented in logarithmic scale in Fig. S3a, which improves the visibility of lower-intensity peaks. It is evident that, unlike surface GaAs QWs,[13] the emission intensity from our surface GaN QWs remains considerably strong even in the absence of a surface barrier. In this logscale plot, the AlGaN spacer emission from all the samples exhibits an almost identical intensity. This confirms that the injection level into the samples is nearly the same, unaffected by the variation of d.

Since the line shape of CL spectra changes across samples, a quantitative comparison of the QWs with varying d requires peak fitting. For these CL spectra, due to the inhomogeneous broadening caused by spatial fluctuations (Sec. 5), the peaks are fitted by Gaussians to extract the peak energy and integrated intensity of the GaN QW and AlGaN spacer emissions (Figs. S3b, S3c). In all the plots depicting the d-dependent CL intensity/energy, the error bars in integrated intensity/peak energy are estimated from the Gaussian fitting and the spatial fluctuation detailed in Sec. 5.

The peak energies of the surface GaN QWs are presented in Table 1. Notably, the peak energy of the AlGaN spacer emission remains nearly constant across all samples. In contrast, the GaN QW peak exhibits a small blueshift for the sample with $d = 1$ nm and an even

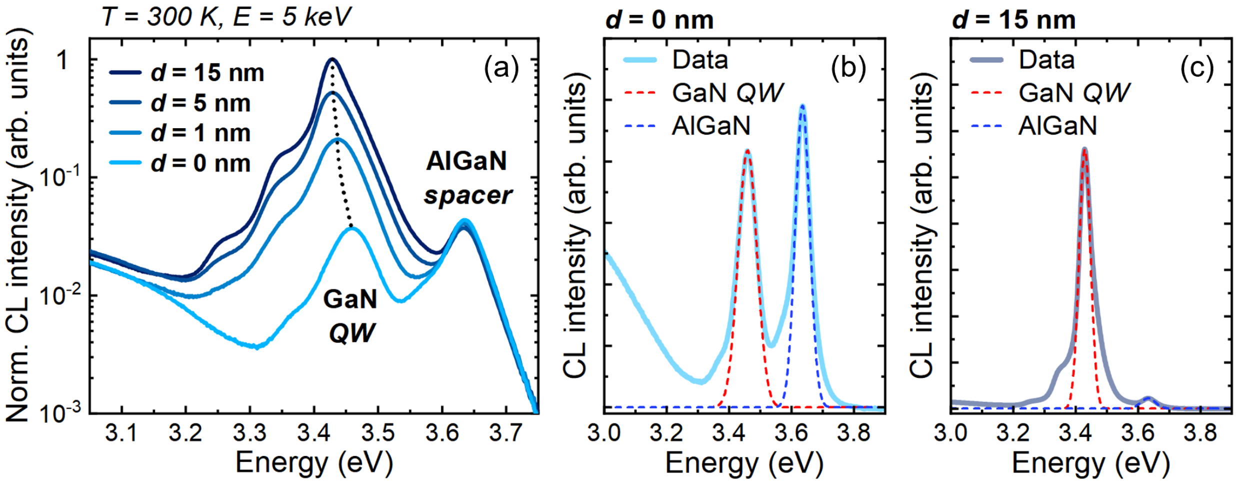

Figure S3: (a) CL spectra of the surface GaN QWs, excited by a 5 keV electron beam at 300 K. The intensity scale is set to logarithmic to enhance the visibility of lower-intensity peaks. (b, c) For each CL spectrum, a Gaussian fit is applied to extract the peak energy and the integrated intensity of the GaN QW and AlGaN spacer emissions. The shoulders on the low energy side of the QW peak correspond to its longitudinal optical (LO) phonon replicas.

Table 1: Peak energies of the GaN QW and AlGaN spacer emissions deduced from the CL spectra of surface GaN QWs with varying surface barrier thickness (d), shown in Fig. S3a.

d	**0 nm**	**1 nm**	**5 nm**	**15 nm**
GaN QW	3.459 eV	3.437 eV	3.430 eV	3.429 eV
AlGaN spacer	3.635 eV	3.635 eV	3.634 eV	3.632 eV

larger blueshift for the uncapped QW ($d = 0$ nm). To gain insights into these observations, we conducted band diagram calculations and analyzed the corresponding confined states using the commercial software, *nextnano*,[14] as shown in Fig. S4a. For these polar surface QWs, the presence of the free surface restricts the electron wavefunction spreading, which leads to enhanced quantum confinement of carriers in the well when d is small. To further analyze the emission energy as a function of d, we compared the calculated interband transition energies with the GaN QW peak energies extracted from CL measurements (Fig. S4b). While the overall d-dependent trends are in agreement, the calculated values for QWs with $d = 0$ and 1 nm are much higher than the experimental data. This disparity arises from the Dirichlet boundary condition applied at the free surface in the simulation, which does not consider the evanescent wave in the vacuum, thus leading to an overestimation of the confinement energy

induced by the free surface. Despite this discrepancy, the overall agreement between the two trends provides a reasonable explanation for the large blueshift observed in the uncapped QW: carrier quantum confinement in this "well" is significantly enhanced by the free surface.

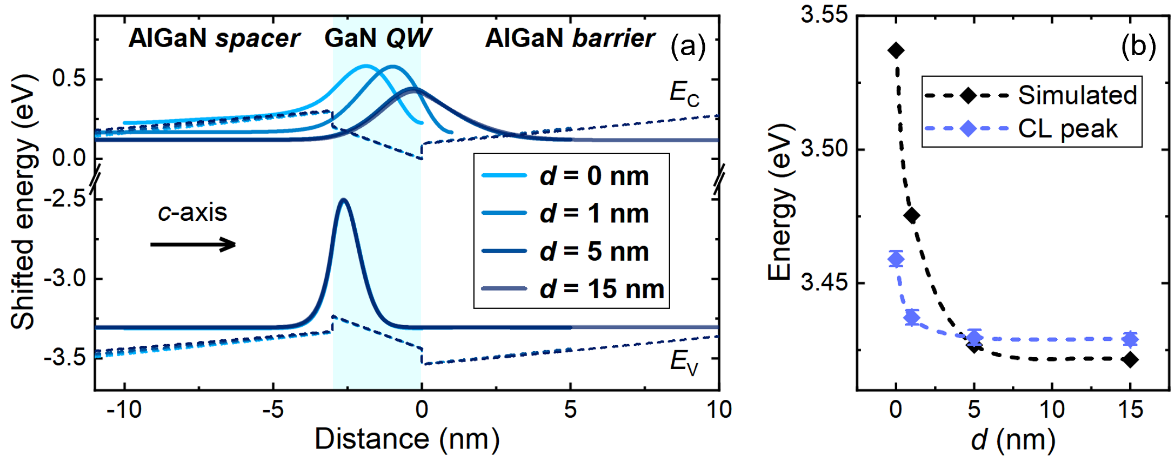

Figure S4: (a) Simulated RT band diagrams of surface GaN/AlGaN QWs with varying surface barrier thickness (d), accompanied by the corresponding wavefunction probability densities of electrons and holes in the QW region. All the wavefunction probability density curves are shifted by the energy of their associated quantized state. E_C and E_V are the conduction band minimum and valence band maximum, respectively. Based on the simulations, (b) the RT interband transition energy is plotted as a function of d (black diamonds), and compared to the CL data (blue diamonds).

In order to investigate the change in the QW emission under different injection levels, we compare the CL spectra with the photoluminescence (PL) spectra of the same samples. The PL spectra were obtained using a continuous wave (cw) laser with a power density of around 16 W/cm^2 at a wavelength of 325 nm. Under this excitation condition, assuming that nearly all carriers relax to the QW and using a carrier lifetime of 1 ns,[11] the estimated carrier density in the QWs is on the order of 10^{10} cm^{-2},[15] which is nearly two orders of magnitude lower than the estimated carrier density in CL (Sec. 2). As depicted in Fig. S5, the CL peaks are generally blueshifted by $\sim$ 20 meV, which can be attributed to a stronger screening of the built-in field in the QW due to the high carrier density, i.e., the quantum-confined Stark effect (QCSE). Interestingly, the line shape of the QW peaks remains nearly identical between CL and PL spectra. If the carrier density in CL were above the critical density of

the Mott transition, a much more asymmetric line shape would be expected due to band filling of the continuum states.[16] Our observation suggests that in the CL measurements, carriers in the QWs predominantly exist as excitons rather than electron-hole pairs.

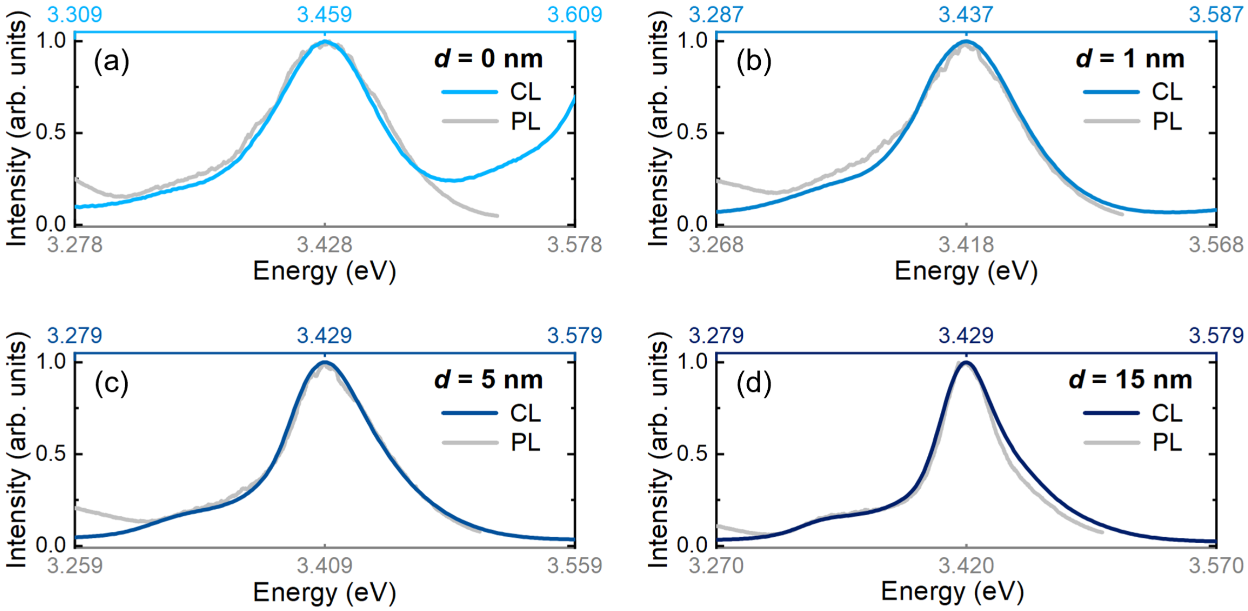

Figure S5: Comparison of the QW emission line shape under different injection conditions: low carrier density ($\sim 10^{10}$ cm^{-2}) in PL and high carrier density ($\sim 10^{12}$ cm^{-2}) in CL at 300 K, for surface GaN QWs with varying barrier thickness: (a) $d = 0$ nm, (b) $d = 1$ nm, (c) $d = 5$ nm, and (d) $d = 15$ nm. The energy axis of the CL spectra is shifted to match the peak energy in the PL spectra while maintaining the same scale.

4. Thickness determination of MoS_2

2D MoS_2 flakes were first obtained by mechanically exfoliating bulk MoS_2 crystals, and deposited onto a Si substrate pre-coated with a 275 nm thick SiO_2 layer. This particular thickness of oxide was chosen to optimize the visibility of ML-MoS_2 under the optical microscope, based on light interference.[17] The flakes of interest were initially identified using optical microscopy (Fig. S6a), and their thickness was subsequently determined by AFM (Fig. S6b). For our MoS_2 samples with lateral thickness variation, we estimated the layer thickness in different regions based on the typical ML-MoS_2 thickness of ~ 0.65 nm. However, it must be acknowledged that in the AFM measurements, ML-flakes on bare substrates

showed a broad distribution in heights, ranging from 0.6 to 0.9 nm,[18] which may be due to the presence of adsorbates beneath the flake or other flake-substrate interactions.[19]

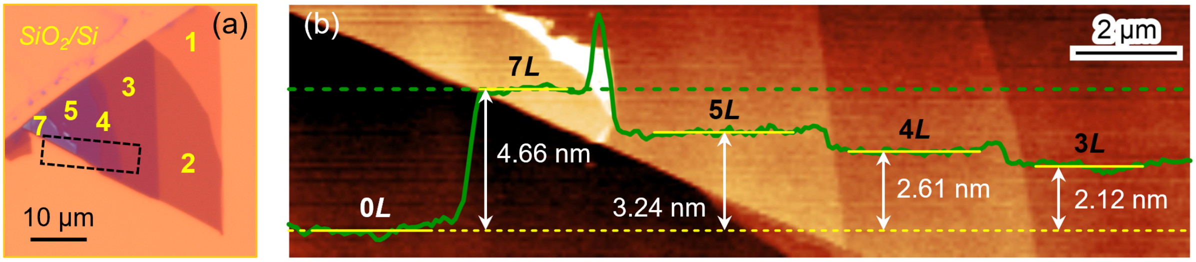

Figure S6: (a) Optical micrograph of the selected MoS_2 flake, deposited on an SiO_2/Si substrate. The numbers in yellow indicate the number of MoS_2 MLs in the corresponding region, determined by AFM and Raman spectroscopy. (b) AFM height image of the rectangular area outlined by the black dashed line in (a). The thickness of each layer is determined from the height profile (green solid curve) taken along the green dashed line in the AFM image. The "nL" labels indicate that the MoS_2 thickness in the corresponding region is n MLs.

To double-check the layer thickness, Raman spectroscopy measurements were carried out in different regions using a cw 532 nm laser in an air ambient environment (Fig. S7a). This method is based on the fact that the Raman frequencies of the E^1_{2g} mode (in-plane opposite vibrations of S and Mo atoms, illustrated in Fig. S7b) and the A_{1g} mode (out-of-plane vibration of S atoms in opposite directions, illustrated in Fig. S7b) are highly sensitive to MoS_2 thickness within the range of $1-4$ MLs.[18] Especially, the difference between the two peaks in ML-MoS_2 generally falls within the range of $18-21$ cm^{-1}, regardless of the laser[20] or substrate[21] used. Therefore, this feature is commonly used to identify ML-MoS_2. In our case, the observed thickness-dependent Raman peaks and the frequency difference between the two peaks (diamonds in Fig. S7c) align well with the reported trend (dashed curves in Fig. S7c),[18] thus corroborating the thickness determined by AFM.

In the end, the thicknesses in regions of $1-3$ MLs were double-checked using AFM and Raman spectroscopy. As depicted in Fig. S8a, Raman results from all samples show good agreement with previous reports.[18,20] Meanwhile, the thicknesses in regions of $4-9$ MLs were double-checked using AFM and optical microscopy.[22,23] Specifically, we computed the normalized optical contrast in regions with varying thicknesses from the grayscale image

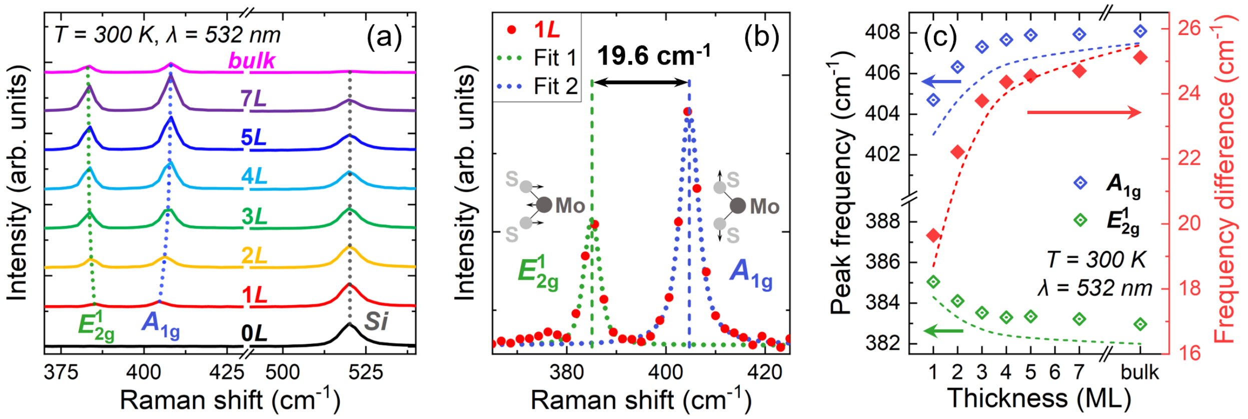

Figure S7: (a) Raman spectra obtained from various regions of the MoS_2 flake illustrated in Fig. S6a. Each spectrum is labeled with the layer thickness (number of MLs, nL) estimated through AFM measurements. A double-Voigt fit is applied to each spectrum to extract the peak frequencies of the E^1_{2g} and A_{1g} Raman modes. (b) In the ML-MoS_2 region (1L), the difference between the two peaks is $\sim$ 19.6 cm^{-1}, which falls within the expected range of ML-MoS_2. (c) The peak frequencies of the E^1_{2g} and A_{1g} Raman modes (left vertical axis) and their difference (right vertical axis) are plotted as a function of layer thickness. Our results (diamonds) are compared with the expected trends (dashed curves).[18]

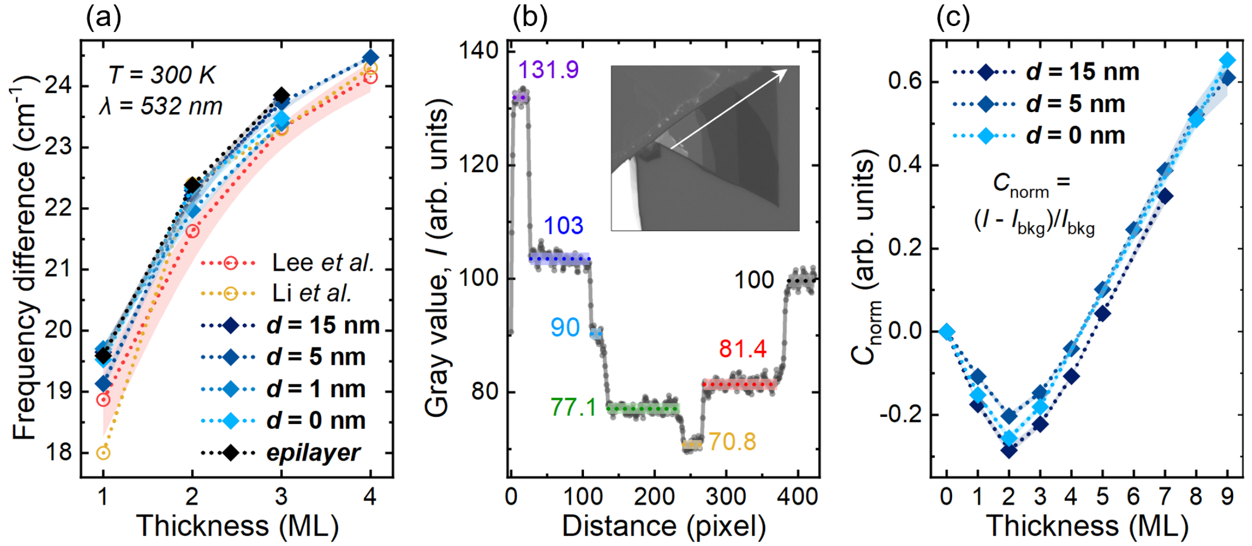

Figure S8: (a) Frequency difference between the E^1_{2g} and A_{1g} Raman modes as a function of layer thickness, compared with values from the literature (Lee *et al.*[18] and Li *et al.*[20]). (b) Brightness profile across an MoS_2 flake (the corresponding optical micrograph is depicted in Fig. S6a), taken along the arrow line in the blue-channel grayscale image of the optical micrograph (inset). (c) Normalized optical contrast (C_{norm}) as a function of MoS_2 thickness for the flakes featuring regions with thicknesses greater than 3 MLs. The normalization was done with respect to the background gray value, I_{bkg}, derived from the uncovered substrate surface. All our data are labeled by the substrate onto which the flake was transferred.

(Fig. S8b), which exhibits consistent linearity for layer thicknesses between 3 and 9 MLs (Fig. S8c).

5. CL data processing

The hyperspectral analysis was conducted using the *hyperspy* package in Python.[24] As depicted in Figs. S9a, S9b, the CL spectrum from an individual pixel exhibits a relatively low signal-to-noise ratio due to the very low exposure time (0.02 s), which is chosen to mitigate potential luminescence quenching due to electron beam exposure-related charge trapping and carbon contamination.[25] This noise level makes accurate spectral fitting challenging. Therefore, we adopt an analytical approach to extract the integrated intensity and peak energy of the GaN QW and AlGaN emissions. First, we compute the average spectra of the entire map and identify points of zero intensity gradient (Fig. S9c). Three points are found, corresponding to the intersections between the defect-related blue luminescence (BL) band,[26] the GaN QW band, the AlGaN band, and the background. Then, for each pixel spectrum, we integrate between these energies to derive the intensity of the GaN QW and AlGaN emissions at that position (Fig. S9b), which generates the corresponding intensity maps (Fig. S9d). To determine the peak energy, we employ a median filter to reduce the intensity noise (Fig. S9b), the peak within the specified energy range is then identified, generating the corresponding energy map (Fig. S9e).

The integrated intensity maps of the GaN QW and AlGaN emissions of all QW samples in this study are presented in Fig. S10, accompanied by the optical micrographs of the corresponding MoS_2 flakes, taken on an SiO_2/Si substrate. The color contrast in the micrographs allows us to identify regions of MoS_2 with different thicknesses.

To extract information from regions with different MoS_2 thicknesses, one might first consider "line" cuts across the regions of interest. However, this approach is challenging for several reasons. Firstly, given the low signal-to-noise ratio in spectra from individual pixels,

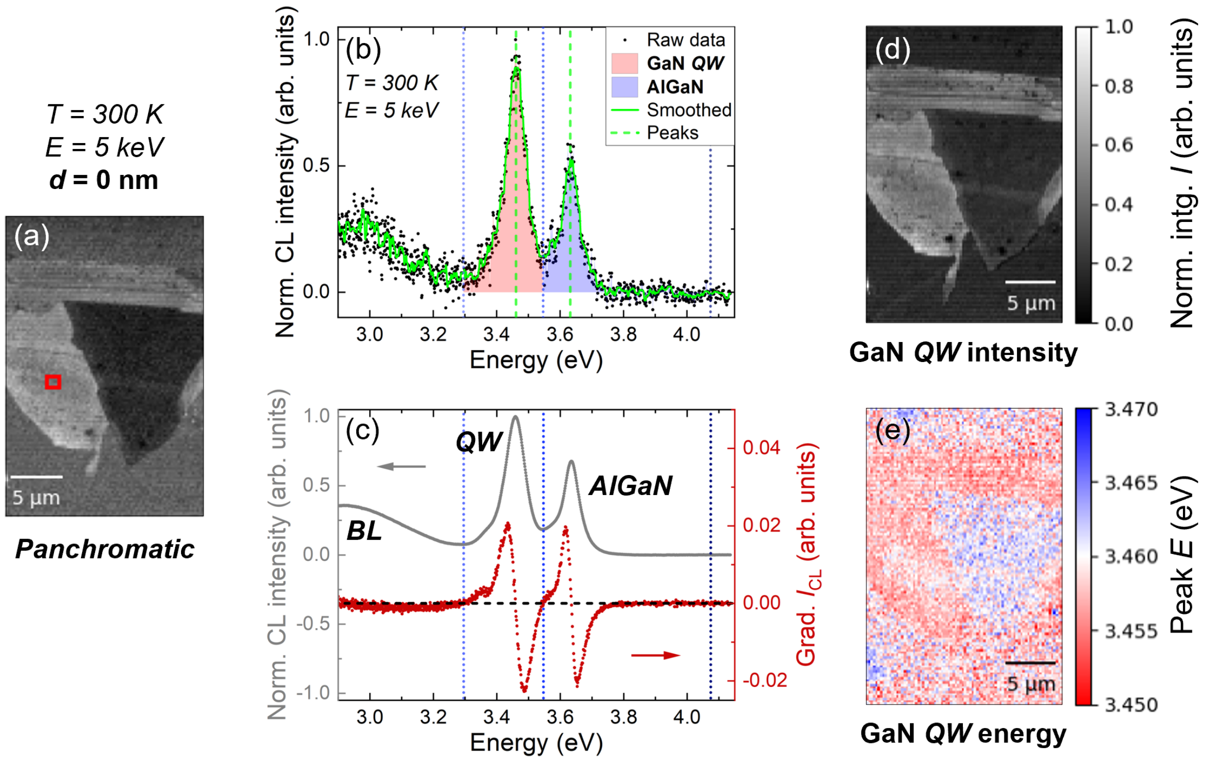

Figure S9: (a) Panchromatic CL map of the uncapped QW ($d = 0$ nm) coated with MoS_2, measured at 300 K using a 5 keV electron beam. (b) Normalized CL spectrum acquired at the pixel represented by the red square in (a). (c) Average spectrum of the whole map (a) and its intensity gradient. Three zero-gradient points, which correspond to the crossovers of different emission bands, are identified. By performing a spectral analysis for all pixels, (d) the integrated intensity map and (e) the peak energy map of the GaN QW emission are generated. All the intensity maps are normalized by their respective maximum and minimum values, and are plotted on a linear intensity scale of $0 - 1$.

discerning an $\sim 10\%$ change in intensity, as reported in Figs. 3b, 3c of the main text, through point-to-point comparison is impractical. Secondly, QW luminescence can be influenced by various factors such as surface morphology, alloy disorder, well thickness fluctuation, and the local quality of the substrate. As shown in Fig. S11, the QW emission intensity extracted from different regions of the bare surface, i.e., without MoS_2 coating, exhibits an $\sim 14\%$ fluctuation in areas of $4.5 \times 4.5\ \mu m^2$, and the mean value may change by $\sim 20\%$ due to the presence of defects (dark spots in CL maps). With such fluctuations, microscale features extracted from a line cut highly depend on the position chosen and thus cannot be representative of the general optical properties. Lastly, although van der Waals (vdW) interactions lead to

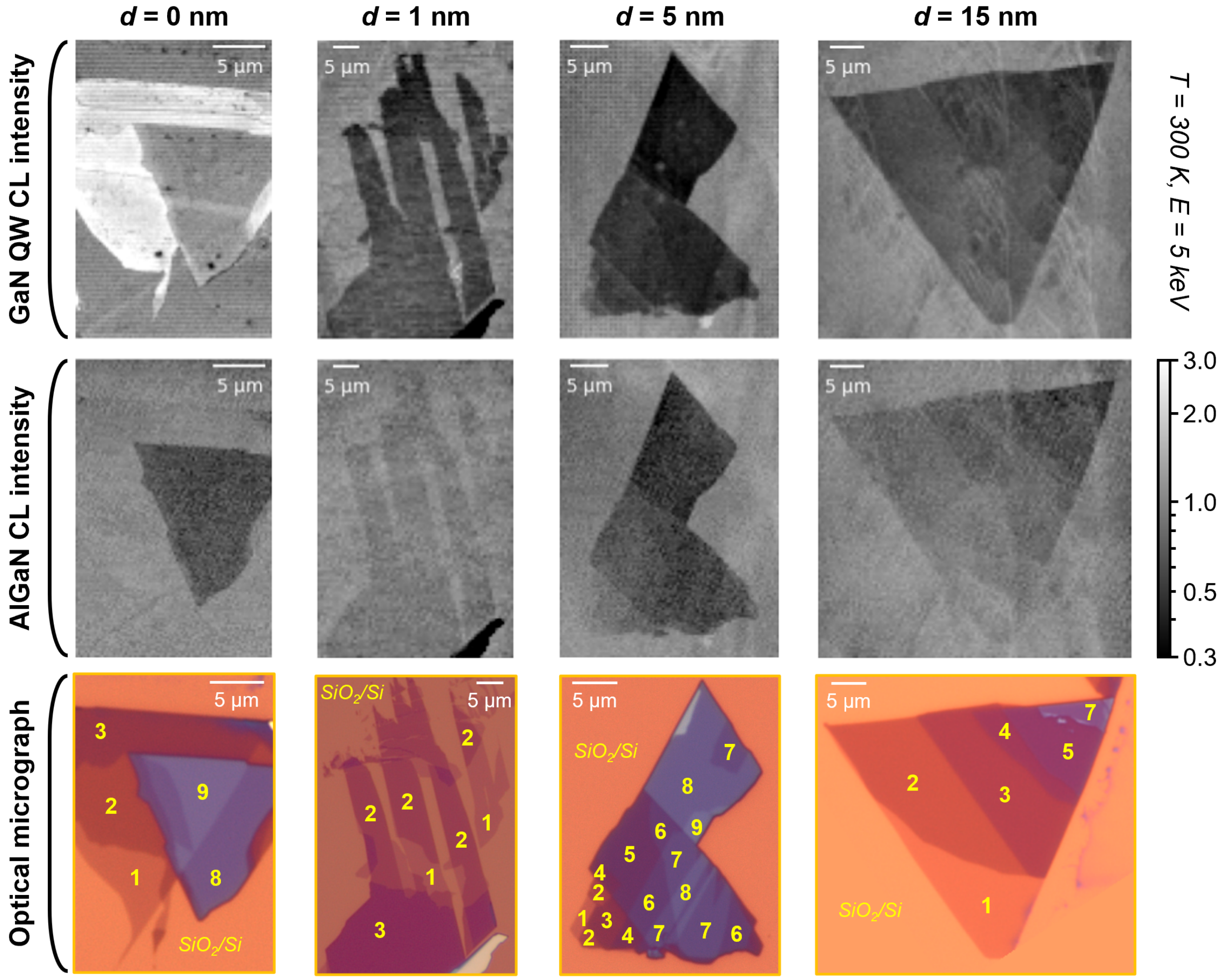

Figure S10: Normalized integrated CL intensity maps of the GaN QW and AlGaN emissions from all the QW samples, acquired with an electron beam energy of 5 keV at 300 K. For each map, the normalization was performed using the average intensity in the region without MoS_2. All CL maps are plotted on a logarithmic intensity scale ranging from 0.3 to 3. Optical micrographs of the corresponding MoS_2 flakes, deposited on an SiO_2/Si substrate, are presented to identify regions of different MoS_2 thicknesses. The numbers in yellow indicate the number of MoS_2 MLs in the corresponding region.

a “self-cleaning” of 2D material interfaces, as they drive small molecules into pockets and leave the rest of the interface atomically clean,[27] the MoS_2-GaN interface might be different: the c-plane GaN surface is polar and is terminated by many dangling bonds, which could potentially help trapping surface adatoms. This, coupled with intrinsic defects present in MoS_2, could also cause spatial fluctuations in CL from the MoS_2-coated regions. Considering all these noises and fluctuations, it is more appropriate to analyze spectra averaged over a

relatively large area rather than relying on point-to-point comparisons.

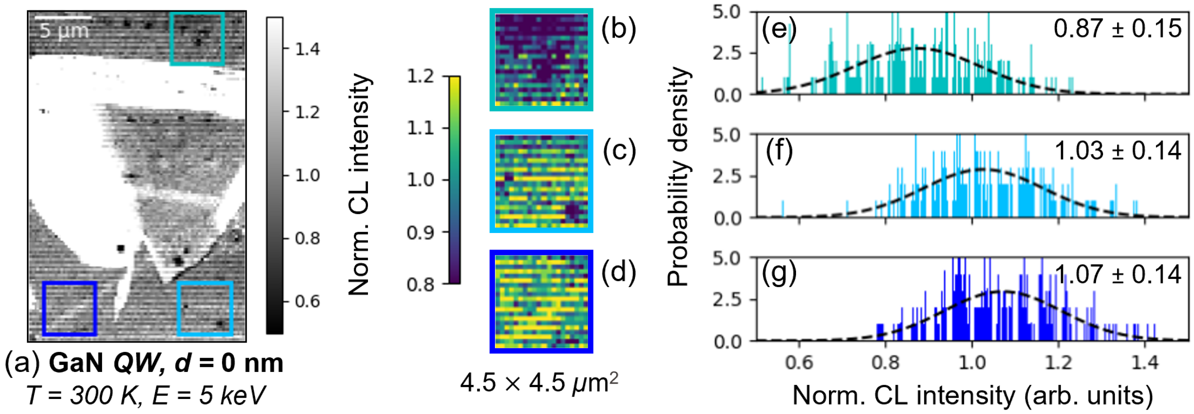

Figure S11: (a) Normalized integrated CL intensity map of the GaN QW emission from the uncapped QW ($d = 0$ nm), acquired with an electron beam energy of 5 keV at 300 K. The normalization is done using the average intensity in the region without MoS_2. (b, c, d) QW intensity maps extracted from different regions of the bare surface (no MoS_2), outlined by blue rectangles in (a). These areas exhibit varying numbers of defects (dark spots in the map), with (b) having the highest and (d) the lowest. Each area measuring 4.5×4.5 μm^2. To enhance the visibility, the colormap and intensity scale of the cropped areas are changed. (e, f, g) Respective intensity histograms of (b, c, d) fitted by a normal distribution, with the mean and standard deviation of the fit expressed in the corresponding plot.

For this purpose, we employed image segmentation on the high-resolution optical micrograph of the flake (Figs. S12a, S12b). This is based on the thickness-sensitive color contrast mentioned in Sec. 4. Consequently, regions with different thicknesses are assigned specific colors, i.e., defined pixel values. The resulting segmented image was then rotated and resized to align it with the contour of the flake in the panchromatic CL map (Fig. S12c). The reshaped image was subsequently cropped and binned to generate a mask for the CL map, ensuring that its image size and number of pixels are the same as the CL map (Fig. S12d). Due to the lower resolution of the CL map, pixels located at the boundaries of the segmented regions exhibit intermediate values after the binning process. To address this, all these new pixel values were converted to (255, 255, 255) (white in color), which represent "dead pixels" and are excluded from further data processing and analysis. For all the CL maps in this study, we generated an associated mask where the color of each pixel represents the number of MoS_2 MLs at that position.

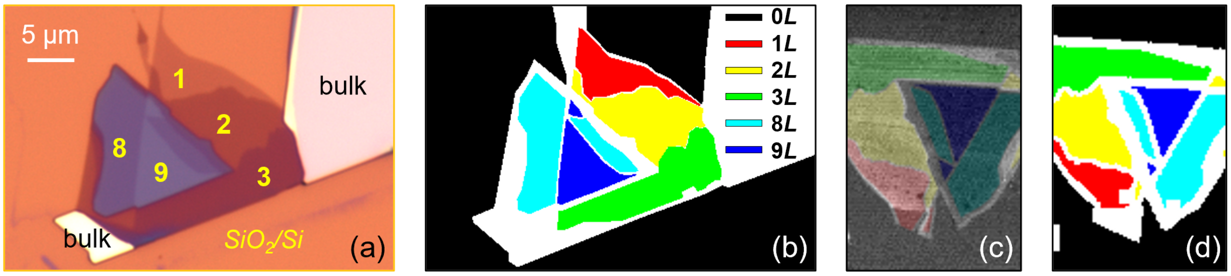

Figure S12: (a) High-resolution optical micrograph of the MoS_2 flake deposited on an SiO_2/Si substrate, which is (b) segmented according to thickness-sensitive color contrast. (c) The segmented image is rotated and resized to match the flake in the CL map. (d) After cropping, pixel binning, and dead pixel identification, a mask of the CL map is generated.

By utilizing the mask shown in Fig. S12d and the corresponding integrated intensity maps (Fig. S10), we extracted the integrated intensities of the GaN QW and AlGaN spacer in regions covered by different MoS_2 thicknesses. The resulting intensity histograms of the two emissions in various regions are depicted in Figs. S13a, S13b. By fitting the histograms with a normal distribution, we computed the mean value and standard deviation in the integrated intensity, which are plotted in the Figs. 3b, 3c of the main text. Interestingly, in Figs. S13c–S13e, there is no clear correlation between the number of pixels and the standard deviation in the corresponding region. This affirms that the estimated standard deviation of the CL intensity is more linked to spatial fluctuations in the sample than a systematic error due to sampling. Notably, the fluctuation is more pronounced in the QW emission (Fig. S13c) compared to that of the AlGaN spacer (Fig. S13d). This is expected due to the significantly smaller thickness of the QW, which amplifies the influence of thickness fluctuations on QW CL intensity. For all comparisons across different QW samples in the study, this standard deviation resulting from spatial fluctuations (σ_{fluct}), combined with the fitting error mentioned in Sec. 3 (σ_{fit}), accounts for the error bar of the integrated intensity/peak energy ($\sigma_{\text{tot}} = \sqrt{\sigma_{\text{fluct}}^2 + \sigma_{\text{fit}}^2}$).

The use of thickness-related masks is crucial to our study. Firstly, these masks enable the calculation of the average background intensity (I_{Bkg}) in different emission maps, which facilitates comparison across different samples. Specifically, the normalized intensity is cal-

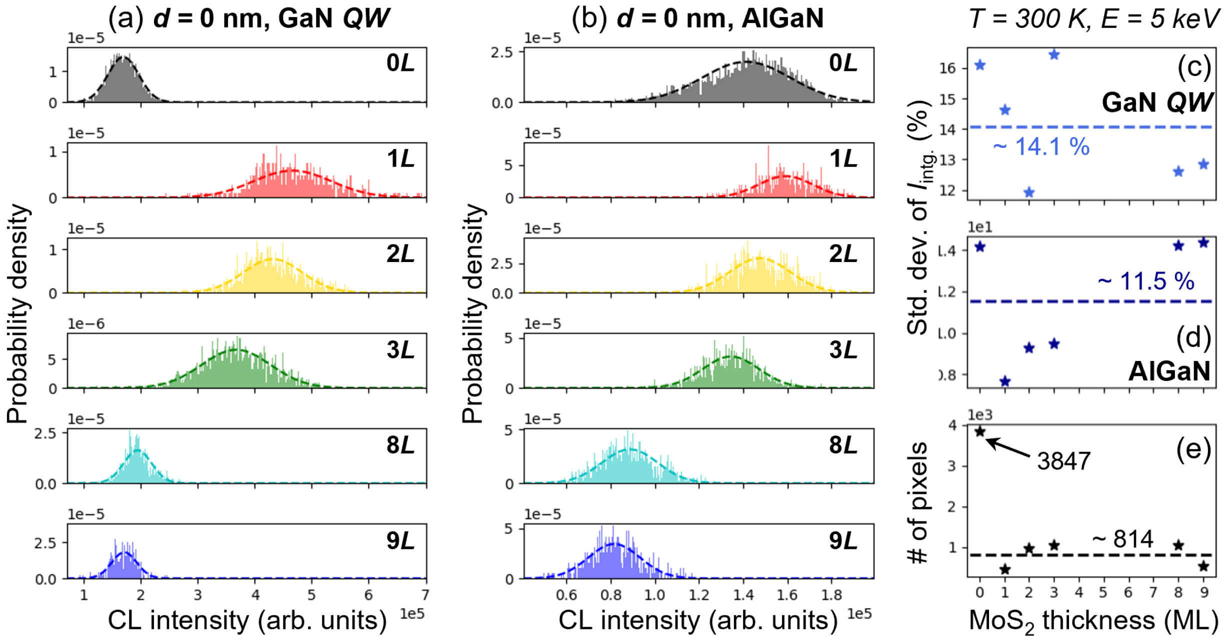

Figure S13: RT histograms of (a) GaN QW and (b) AlGaN spacer integrated intensities extracted from different regions of the uncapped GaN QW, as defined in Fig. S12d. Each subplot is labeled with the number of MoS_2 MLs in the corresponding region, and the dashed line represents the normal fit of the histogram. The standard deviation in intensity deduced from the fit is plotted as a function of MoS_2 thickness for (c) GaN QW and (d) AlGaN spacer emissions, respectively. (e) The number of pixels in different regions is also plotted as a function of MoS_2 thickness. The horizontal dashed lines in (c, d, e) correspond to the average level of the data in the plot, with the value next to them. In (e), the outlier (number for the uncoated region, $0L$) is not considered for the average.

culated using $I_{\text{norm}} = I/I_{\text{Bkg}}$, so that the average background intensity is set to 1 for all maps (Fig. S10). In this way, the contrast observed in each map mainly represents the impact of MoS_2 on the optical properties of the surface GaN QWs, allowing direct comparisons between different maps. Secondly, instead of comparing the intensity/energy maps that also reflect spatial variations related to the surface morphology and structural fluctuations of the sample, we can focus on the spectral domain by comparing the average spectra obtained from regions coated with MoS_2 of different thicknesses, as depicted in Fig. S14. The advantage of this analysis is twofold. On the one hand, the average spectrum captures the general properties of the vdW interface effect associated with MoS_2 thickness and effectively minimizes the impact of microscale fluctuations arising from surface morphology or other factors that are

not the main focus of this study. On the other hand, by averaging a large number of spectra, the resulting spectrum has a higher signal-to-noise ratio. This facilitates quantitative analyses, including the peak identification (e.g., zero-phonon line and LO phonon replicas of the GaN QW emission, depicted in Fig. 1b of the main text) and the precise fit of the peaks of interest (Figs. S3b, S3c), which serves for comparisons across different QW samples (see Fig. 5a in the main text). It is worth noting that for $d = 1$ nm (Fig. S14b), the peak intensity of the GaN QW is higher when coated by 3 MLs of MoS_2 compared to those coated by 1 or 2 MLs, unlike the behavior observed in other samples (Figs. S14a, S14c, S14d). The

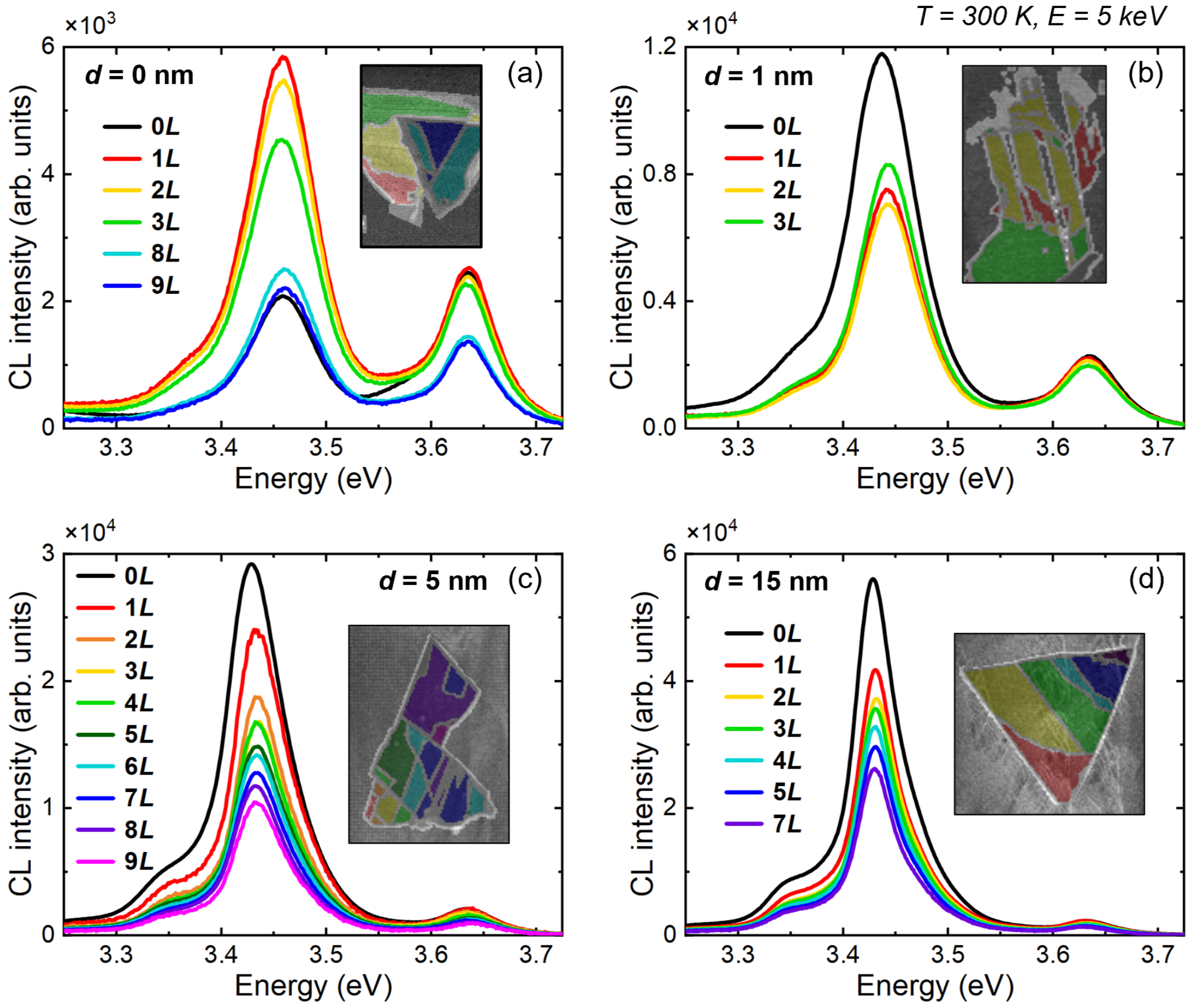

Figure S14: Average RT CL spectra extracted from regions with different MoS_2 thicknesses for QWs with (a) $d = 0$ nm, (b) $d = 1$ nm, (c) $d = 5$ nm and (d) $d = 15$ nm. The corresponding regions are shown in the inset.

origin of this discrepancy is unclear, but the integrated QW intensity extracted from this spectrum (d = 1 nm, $3L$) is still consistent with the model used to fit the data shown in Fig. 5a. Therefore, this discrepancy is not strongly influencing the surface recombination mechanism, which is the main focus of our study. Overall, the use of thickness-related masks enables equivalent data normalization, spectral analysis, and quantitative calculations, which improves the interpretability and reliability of our results.

6. Spectral absorptance of ML-MoS_2

The spectral absorbance of ML-MoS_2 (A) has been extracted from independent studies of MoS_2 on sapphire (Dumcenco *et al.*[28]) and PDMS (Castellanos-Gomez *et al.*[29]) substrates, measured at RT. To compute the spectral absorptance (a), we apply the relation by definition: $a = 1 - 10^{-A}$. In Fig. S15, the comparison of the two curves reveals that the ML-MoS_2 absorptance does not vary significantly with the substrate at energies far from the $\boldsymbol{A}$, $\boldsymbol{B}$, and $\boldsymbol{C}$ peaks. Considering that the emission of our QWs peaks at the valley between the $\boldsymbol{C}$ and $\boldsymbol{D}$ bands, it is reasonable to utilize the reported value as a reference. Since only the measurement conducted by Dumcenco *et al.* covers the spectral range of interest for our study, their result is used as reference for all the absorptance values considered in our study. The

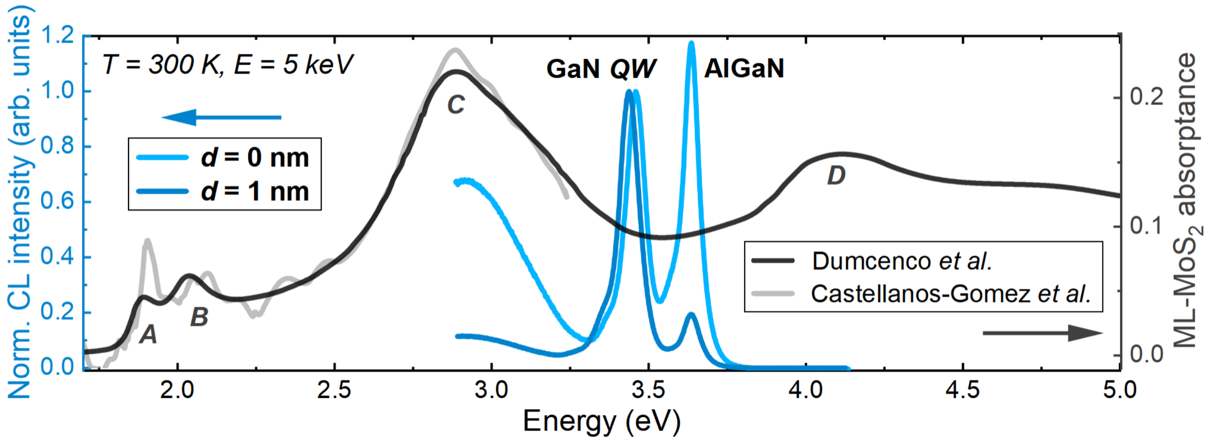

Figure S15: RT spectral absorptance of ML-MoS_2 derived from different studies[28,29] and CL spectra of the QWs with $d = 0$ and 1 nm, measured at 300 K using a 5 keV electron beam.

QW peak energy, that of the AlGaN layer, and their corresponding absorptance for the two QW samples are listed in Table 2. Given that the change in the QW peak energy caused by varying d is negligible compared to the broad MoS_2 absorptance bands, and that the shift in energy for the $Al_{0.1}Ga_{0.9}N$ alloy versus GaN is marginal, the ML-MoS_2 absorptance remains around $9 - 10\%$ for all the emission peaks discussed in this study.

Table 2: Peak energies of interest and their corresponding ML-MoS_2 spectral absorptances.[28]

**Peak**	$d = 0$ **nm, QW**	$d = 0$ **nm, AlGaN**	$d = 1$ **nm, QW**
Energy	3.459 eV	3.635 eV	3.437 eV
Spectral absorptance	9.4%	9.4%	9.6%

7. MoS_2 on bulk GaN epilayer

To confirm the hypothesis of the surface effect, we deposited an MoS_2 flake of $1 - 3$ MLs on a bulk GaN epilayer, identical to the GaN buffer used to grow the QWs (Sec. 1). CL measurement was performed on this flake at 300 K using 5 keV excitation. The resulting GaN intensity map is normalized (inset of Fig. S16a) and compared to the normalized GaN QW intensity map of the uncapped GaN QW ($d = 0$ nm) coated with 2D MoS_2 (inset of Fig. S16b). Through normalization, both maps have the same average background intensity, i.e., 1. Upon comparison, it is evident that both maps demonstrate enhanced GaN emission in the presence of MoS_2. However, the enhancement is obviously stronger for the uncapped GaN QW emission than for the GaN epilayer emission. To quantitatively visualize this difference, average CL spectra of the bare region ($0L$) and the region coated with ML-MoS_2 ($1L$) are compared. For the GaN epilayer, the emission is enhanced by ML-MoS_2, with a peak intensity ratio of ~ 1.4 (Fig. S16a). In contrast, for the uncapped QW, only the GaN QW emission is enhanced, but with a higher peak intensity ratio of ~ 2.8 (Fig. S16b). This discrepancy in MoS_2-induced intensity enhancement between the two cases is consistent with the surface passivation effect. In the uncapped QW, the detected GaN emission comes solely

from the surface region, whereas in the GaN epilayer, the emission arises from both surface and bulk regions. As a result, the enhancement is much greater in the former case due to the direct influence of surface passivation on the carrier recombination process.

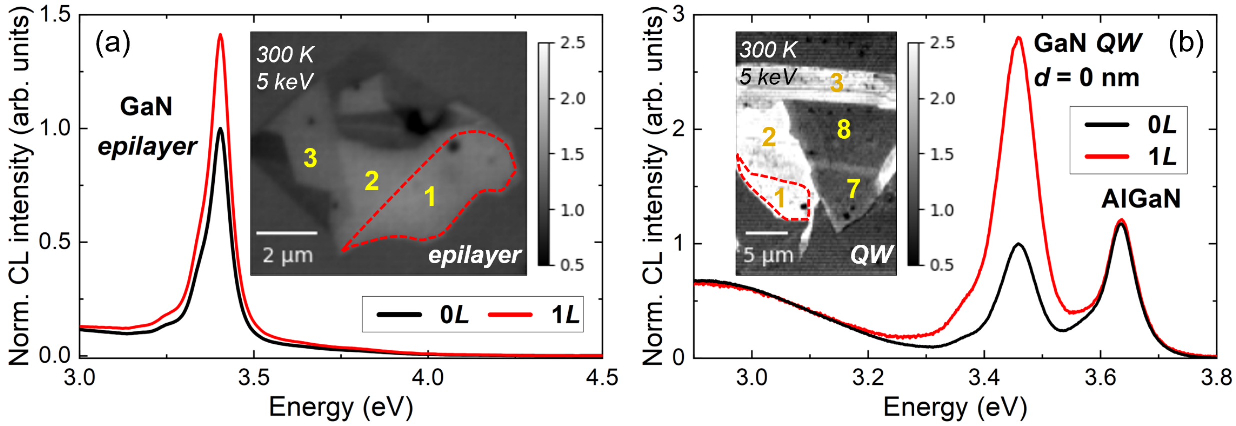

Figure S16: Average RT CL spectra of the background emission (in the absence of MoS_2, $0L$) and the emission from the region covered by ML-MoS_2 ($1L$) extracted from (a) the GaN epilayer and (b) the uncapped GaN QW ($d = 0$ nm), both coated with an MoS_2 flake. The corresponding integrated GaN CL intensity maps are shown in the insets, with the $1L$ region indicated by the red dashed line. These maps are normalized by their average background intensity, and plotted on a linear intensity scale of $0.5 - 2.5$.

For validation of the repeatability and reliability of our results, another 1 ML thick MoS_2 flake was deposited on the GaN epilayer. A CL map was then performed specifically on this coated region (Fig. S17). The results again showed a strong enhancement, with a consistent ~ 1.4 times increase in intensity as observed with the other flake (Fig. S16a). Therefore, we consider our results to be repeatable and reliable.

The increase in surface GaN emission observed when the sample is coated with MoS_2 could be attributed to a passivation of surface states (SSs) caused by charge transfer between the two materials, resulting from the type-II band alignment between MoS_2 and GaN: the unintentionally doped GaN (u-GaN) surface exhibits an upward surface band bending due to the presence of SSs (Fig. S18a); with MoS_2 deposited on the surface, charge transfer between the two materials leads to a downward band bending in u-GaN (Fig. S18b).[30,31] Consequently, SSs in GaN become occupied and are no longer capable of trapping electrons in the surface region, therefore, enhancing surface emission.

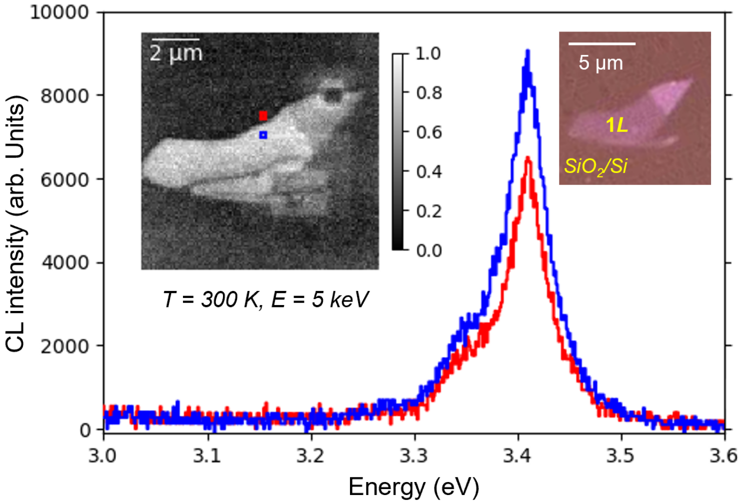

Figure S17: Pixel-to-pixel comparison of RT CL spectra of the background emission (in the absence of MoS_2, red curve) and the emission from the region covered by 1 ML MoS_2 (blue curve), extracted from the GaN epilayer coated with an MoS_2 flake. The peak-to-peak intensity ratio between the two curves is ~ 1.4. The corresponding GaN CL peak intensity map is shown in the inset (left), with the two pixel positions indicated by the colored squares. The map is normalized by its maximum and minimum values, and plotted on a linear intensity scale of $0-1$. The optical micrograph of the flake, taken on an SiO_2/Si substrate, is also shown in the inset (right).

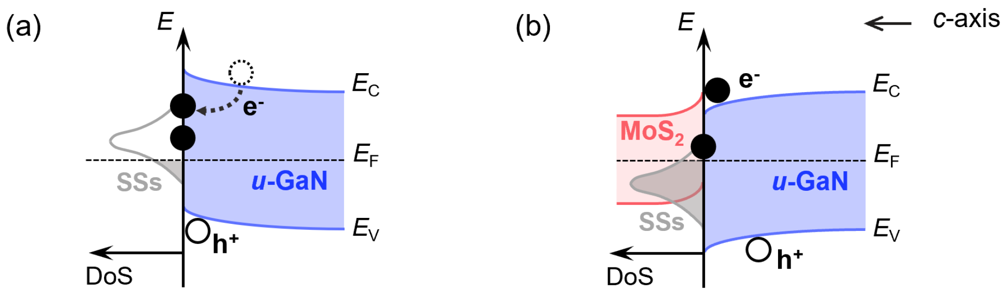

Figure S18: Schematic surface band bending of (a) bare c-plane u-GaN and (b) c-plane u-GaN coated with MoS_2 after interfacial charge transfer.[30,31] E_F is the Fermi level. E_C and E_V are the conduction band minimum and valence band maximum, respectively. The density of states (DoS) of SSs in u-GaN is depicted with a Gaussian distribution in both situations, with the grey shaded part representing occupied states.

8. Possible surface passivation mechanisms

Interference: similar refractive indices

The refractive indices of MoS_2, GaN, and $Al_{0.1}Ga_{0.9}N$ at 360 nm, i.e., around the peak emission wavelength of the uncapped GaN QW, are presented in Table 3. Based on these

Table 3: RT refractive indices of various materials relevant to this study, measured at the wavelength of the uncapped QW, i.e., $\sim$ 360 nm. The refractive index of $Al_{0.1}Ga_{0.9}N$ was estimated through linear interpolation between GaN and AlN.

**ML-MoS$_2$**[32]	**GaN**[33]	**AlN**[34]	**$Al_{0.1}Ga_{0.9}N$**
2.18	2.59	2.20	2.55

values, we can compute the normal reflectance at the interfaces using the Fresnel equation: $R = (\frac{n_1-n_2}{n_1+n_2})^2$, where n_1 and n_2 denote the refractive indices of the two media in contact at the interface. The result yields $R = 0.007$ for the MoS_2/GaN interface and $R = 6 \cdot 10^{-5}$ for the GaN/$Al_{0.1}Ga_{0.9}N$ interface, ruling out the possibility of strong interference effects in our MoS_2/GaN/AlGaN structure.

Quantum-confined Stark effect: no significant energy change

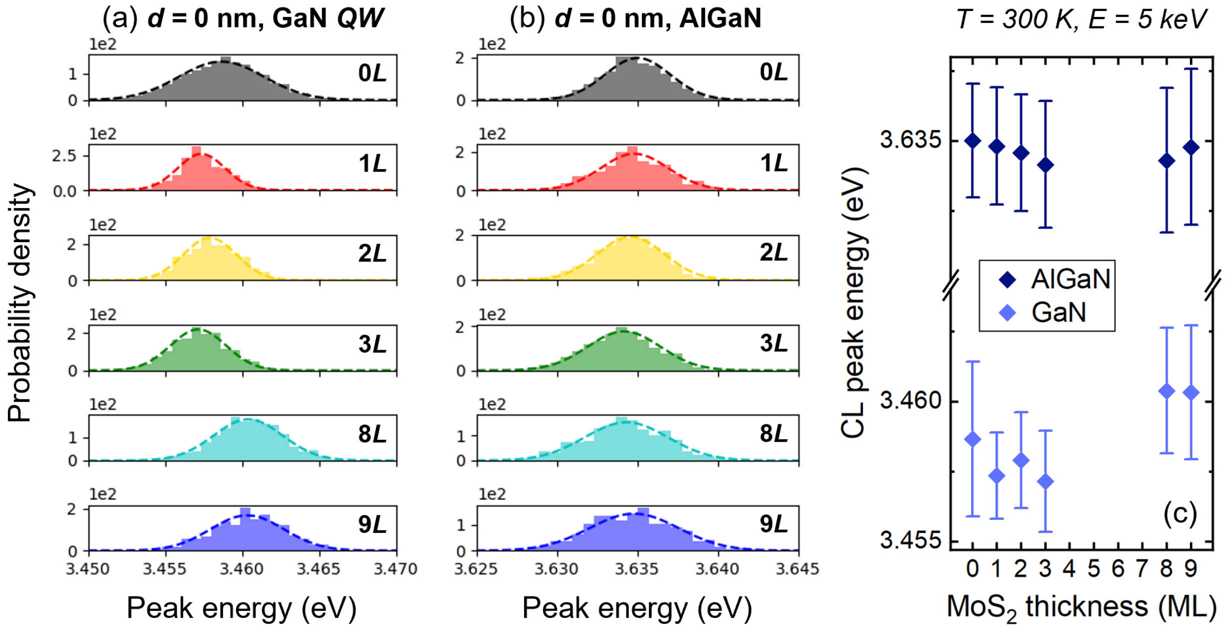

Figure S19: Histograms of (a) GaN QW and (b) AlGaN spacer RT peak energies extracted from different regions as defined in Fig. S12d. Each subplot is labeled with the number of MoS_2 MLs in the corresponding region, and the dashed line represents the normal fit of the histogram. From the fits, (c) the mean peak energies with their standard deviation are plotted as a function of MoS_2 thickness for the GaN QW and AlGaN spacer emissions. Compared to the standard deviation induced by spatial fluctuations, no significant energy change is induced by MoS_2 deposited on the uncapped QW.

9. Degradation of MoS_2 under electron beam irradiation

The degradation of MoS_2 under electron beam irradiation was initially observed on the reference sample, i.e., the GaN epilayer. In the first secondary electron (SE) image taken on this sample (Fig. S20a), the flake exhibited uniform intensity, indicating a smooth morphology, across regions of different MoS_2 thicknesses. However, in the SE image captured after many scans (Fig. S20b), the ML-MoS_2 region ($1L$) displays an evident degradation, which is less prominent in the 2-ML ($2L$) region and not visible in the 3-ML ($3L$) region, highlighting the sensitivity of ML-MoS_2 to the environment. It is important to note that the spatial resolution of the two scans differs, as a lower beam energy was deliberately used to probe the surface morphology for the last scan. Additional evidence of MoS_2 degradation was

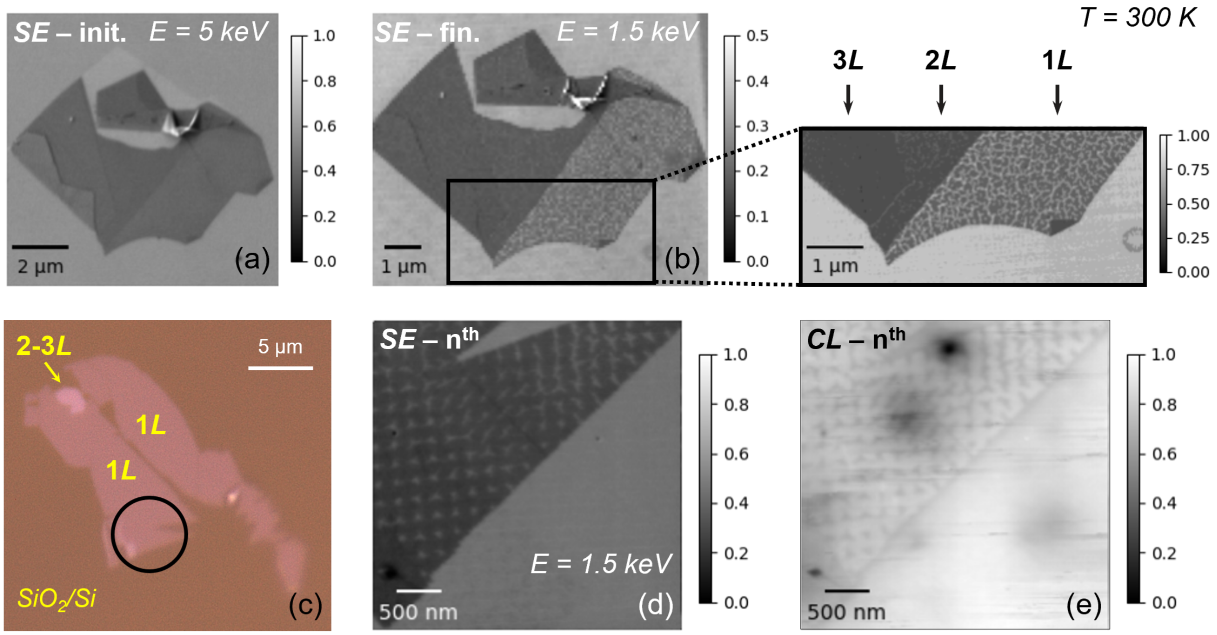

Figure S20: SE images of the MoS_2 flake deposited on the GaN epilayer captured (a) during the first scan and (b) after many scans. The first scan was conducted using the conventional beam energy for CL measurements, 5 keV, while the energy was reduced to 1.5 keV for the last scan to enhance spatial resolution. (c) Optical micrograph of an ML-MoS_2 flake taken on the SiO_2/Si substrate. This flake was then transferred onto an AlGaN surface. In the region indicated by the black circle in (c), an SE image and a panchromatic CL intensity map were taken simultaneously after a few scans. For improved resolution, the beam energy was also set to 1.5 keV. All measurements were performed at 300 K. All the SE images, as well as the CL intensity map, are normalized by their respective maximum and minimum values.

observed on another ML-MoS_2 flake (Fig. S20c) deposited on an InGaN QW, also capped by $Al_{0.1}Ga_{0.9}N$. High-resolution SE imaging was conducted just after a few scans (Fig. S20d), accompanied by the simultaneous acquisition of a CL map (Fig. S20e). Notably, unlike the uncoated region, a distinct pattern is observed on the MoS_2-coated area in the SE image (Fig. S20d). The spacing of the bright spots in this pattern aligns with the map resolution of the previous scans, i.e., the step size of the scanning electron beam. In the corresponding panchromatic CL intensity map (Fig. S20e), a similar pattern is observed on the MoS_2-coated region, proving that this degradation of MoS_2 is impacting the signal emitted from the III-nitride sample underneath. Therefore, the reduction in the MoS_2-induced enhancement of the surface emission, observed in the second scan of the uncapped GaN QW ($d = 0$ nm) (Fig. 4b in the main text), can be attributed to the degradation of MoS_2, particularly in the ML-MoS_2 region, under electron beam irradiation.

10. Model for the d-dependent GaN QW intensity

In Fig. 1b of the main text, the integrated GaN QW intensity is plotted against the surface barrier thickness, d, clearly demonstrating a nonlinear increase with increasing d. However, according to our simulation (Fig. S4a), for larger d, the electron-hole wavefunction overlap is smaller, thus the radiative recombination rate should be lower due to the QCSE. If the weight of non-radiative recombination were the same for all the samples, the QW emission intensity should decrease with increasing d. Therefore, the observed increase in intensity is dominated by a reduction in non-radiative recombination rate.

To model this d-dependent GaN QW intensity, we have to account for non-radiative recombination of carriers in the QWs due to non-radiative recombination centers (NRCs) located in the near surface region, which necessitates an overlap of electron and hole wavefunctions with the corresponding defects. Let us first consider the spreading of carrier wavefunctions in the QWs. The effective barrier height (V_{eff}) for electrons (holes) in the QWs is

approximately equal to the conduction (valence) band offset, ΔE_{C} (ΔE_{V}), between the GaN QW and the $Al_{0.1}Ga_{0.9}N$ barrier. This value can be estimated by considering the bandgap (E_{g}) at 300 K of GaN (3.42 eV[10]) and $Al_{0.1}Ga_{0.9}N$ (3.64 eV[10]), along with the reported 75:25 ratio of conduction-to-valence band offsets for GaN/AlGaN heterostructures:[35] $\Delta E_{\mathrm{C}} = 0.75\Delta E_{\mathrm{g}} = 165$ meV, $\Delta E_{\mathrm{V}} = 0.25\Delta E_{\mathrm{g}} = 55$ meV. The one-dimensional Schrödinger equation for a carrier in the barrier can be written in the form:

$$\frac{d^2}{dx^2}\Psi(x) = \frac{2m^*}{\hbar^2}V_{\mathrm{eff}}\Psi(x) = \kappa^2\Psi(x) , \qquad \text{where} \quad \kappa^2 = \frac{2m^*}{\hbar^2}V_{\mathrm{eff}} . \tag{3}$$

Here $\hbar$ is the reduced Planck's constant and m^* is the effective mass of the carrier (electron or hole) in $Al_{0.1}Ga_{0.9}N$. The solution of this equation is that of an evanescent wave: $\Psi(x) = \Psi_0 e^{-\kappa x}$, with Ψ_0 a constant coefficient. The effective masses of electrons and holes in GaN, along the growth direction (c-axis), are 0.19 m_0 and 1.88 m_0 (m_0 is the electron rest mass), respectively.[36] Since the Al content in the barrier is only 10%, we use these values as the effective masses in AlGaN. This results in $\kappa \approx 0.9$ nm^{-1} for electrons and $\kappa \approx 1.6$ nm^{-1} for holes, corresponding to a wavefunction spreading of ~ 1 nm into the barrier, which is equal to or smaller than the surface barrier thickness of the QWs (d). Therefore, the penetration of carriers from the well into the barrier is not the main origin of the d-dependent QW intensity.

Furthermore, in our CL measurements, the built-in field in the QW should not be completely screened by the carrier density of $\sim 10^{12}$ cm^{-2} (Sec. 3).[16] To validate this point, we conducted band diagram simulations using a one-dimensional Schrödinger-Poisson solver.[16] The strength of the intrinsic built-in field in the $GaN/Al_{0.1}Ga_{0.9}N$ QW is set to the reported value of 570 kV/cm,[37] and the carrier density in the QW (n_{QW}) is varied from $1 \cdot 10^{10}$ cm^{-2} to $1 \cdot 10^{12}$ cm^{-2}, corresponding to the range of the injection levels in the PL and CL measurements (Sec. 3). As depicted in Fig. S21, for a carrier density $n_{\mathrm{QW}} = 1 \cdot 10^{12}$ cm^{-2}, which corresponds to the estimated injection level in our CL measurements, the built-in field in the well is significantly screened compared to the case of $n_{\mathrm{QW}} = 1 \cdot 10^{10}$ cm^{-2}. This reduction

in the strength of the electric field is accompanied by an increase in the interband transition energy of 25 meV, which is close to the QCSE-related blueshift observed between the PL and CL peak energies (Fig. S5). Therefore, we used the wavefunction simulated with $n_{\mathrm{QW}} = 1 \cdot 10^{12}$ cm^{-2} to estimate the probability for holes to be trapped by surface defects through tunneling across a 1 nm thick $Al_{0.1}Ga_{0.9}N$ barrier in our CL measurements:

$$P_{\mathrm{tunneling}}(d) \propto \int_{d}^{\infty} \Psi_{\mathrm{h}}^{2}(x)\mathrm{d}x\ , \tag{4}$$

where Ψ_{h} is the hole wavefunction, with x the distance measured along the c-axis. Here, $x = 0$ is defined at the QW-surface-barrier interface. At $d = 1$ nm, the estimated probability is $\sim 3 \cdot 10^{-7}$, which suggests that after 1 nm, holes in the QW are entirely separated from the surface. Consequently, excitons in the well are no longer influenced by the surface when $d \geq 1$ nm. The increase in QW intensity observed as d changes from 1 to 15 nm (Fig. S22) is primarily attributed to the spatial distribution of NRCs, likely divacancies, in the surface region.

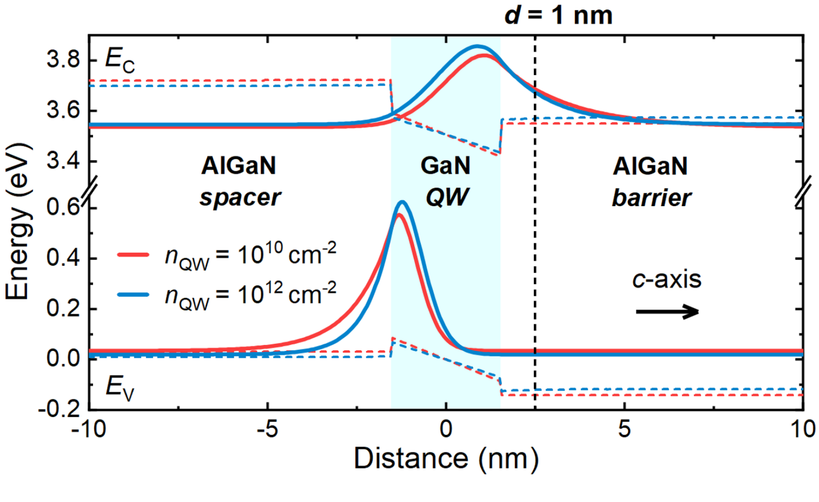

Figure S21: Simulated band diagrams of a single $GaN/Al_{0.1}Ga_{0.9}N$ QW with varying carrier densities in the well (n_{QW}), accompanied by the corresponding wavefunction probability densities of electrons and holes in the QW region. All the wavefunction probability density curves are shifted by the energy of their associated quantized state. E_{C} and E_{V} are the conduction band minimum and valence band maximum, respectively.

It is reported that nitrogen vacancies (V_{N}) and/or V_{N}-related complexes are present in

the surface region of n-type GaN, serving as NRCs that can attenuate the luminescence intensity.[38,39] The concentration of these point defects (PDs) diminishes gradually from the surface to the bulk,[38] potentially attributed to the segregation of V_N in (Al)GaN.[40,41] Therefore, we assume that the PD density at a position d from the surface can be expressed as:

$$[\mathrm{PD}](d) = \theta_0 \cdot e^{-d/L_{\mathrm{eff}}} , \tag{5}$$

with θ_0 the PD density at the free surface ($d = 0$ nm), and L_{eff} a phenomenological parameter accounting for the spatial spreading of PDs in the (Al)GaN surface region. The measured QW emission intensity is correlated to the internal quantum efficiency of the sample, which is defined as the ratio between the radiative recombination rate (R_{r}) and the effective recombination rate (R_{eff}). Given that these samples possess similar structures and were grown under identical conditions, and that their difference in quantum confinement has a negligible impact on the emission, as previously discussed, it is reasonable to assume that R_{r} in these QWs remains constant under the high injection conditions used for CL measurements. Subsequently, the QW intensity should be inversely proportional to R_{eff}:

$$I_{\mathrm{QW}} \propto 1/R_{\mathrm{eff}} , \qquad \text{with} \quad R_{\mathrm{eff}} = R_0 + R_{\mathrm{NR,\,PD}} , \tag{6}$$

where R_0 represents the effective recombination rate in the absence of PDs, i.e., QWs with very large d. $R_{\mathrm{NR,\,PD}}$ denotes the non-radiative recombination rate associated with PDs, therefore $R_{\mathrm{NR,\,PD}} \propto [\mathrm{PD}]$. Given that all the samples exhibit a similar structure and comparable material quality, we can treat R_0, θ_0 and L_{eff} as constants independent of the variable d. Subsequently, by combining Eqs. 5 and 6, we can model the d-dependent QW intensity using the following formula:

$$I_{\mathrm{QW}}(d) = \frac{1}{1 + A \cdot e^{-d/L_{\mathrm{eff}}}} , \qquad \text{where} \quad \lim_{d \to \infty} I_{\mathrm{QW}} \to 1 . \tag{7}$$

Here A is related to the non-radiative recombination rate at the surface. It is important to note that all the QW intensities in Fig. S22 are normalized by their corresponding values at $d = 15$ nm to correct them from MoS_2 absorption. However, this normalization does not imply that at $d = 15$ nm, the PDs no longer influence the emission. Therefore, we should fit the data with an additional parameter A' to account for this correction:

$$I_{\text{norm}}(d) = \frac{1}{A' + A \cdot e^{-d/L_{\text{eff}}}} \,. \tag{8}$$

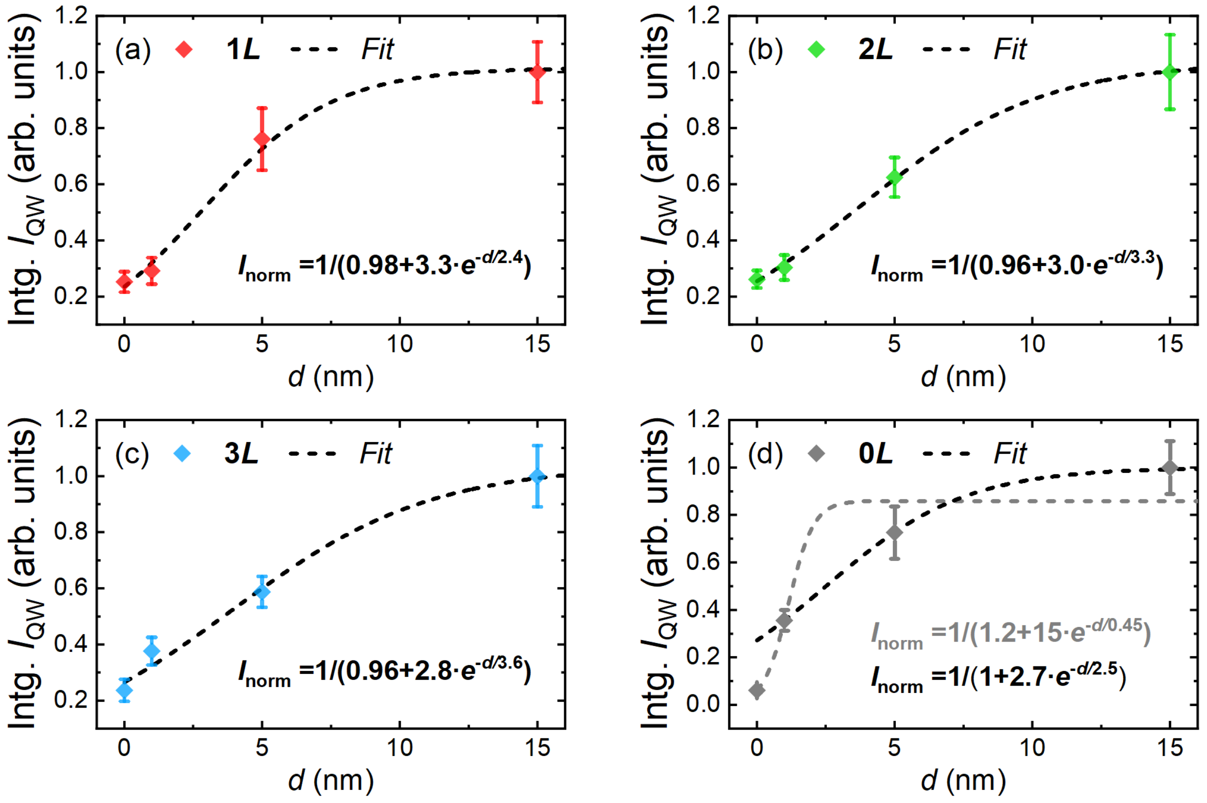

Figure S22: d-dependent integrated QW CL intensity, excited by a 5 keV electron beam at 300 K, extracted from regions coated by MoS_2 of (a) 1 ML, (b) 2 MLs and (c) 3 MLs, fitted by Eq. 8. (d) The data from regions without MoS_2 coating ($0L$) are also fitted by Eq. 8 (gray dashed curve), which fails to capture the QW intensity variation between $d = 5$ nm and 15 nm. They are then fitted with parameter A' fixed to 1 and excluding the outlier at $d = 0$ nm (black dashed curve), which aligns well with the trend. The expressions of the fits are shown in the corresponding plot, and their detailed results are summarized in Table 4.

Eventually, Eq. 8 fits well the d-dependent integrated QW CL intensity extracted from

regions coated by MoS_2 of $1-3$ MLs, with $A' \approx 1$ for all these cases (Figs. S22a–S22c), indicating that the QW of $d = 15$ nm is nearly free from PDs. However, it fails to adequately describe the data for the bare surface without coating (Fig. S22d). Conversely, when excluding the point at $d = 0$ nm and fitting with A' fixed to 1 (due to the limited number of data points), the model performs well (Fig. S22d). Overall, except for the point at $d = 0$ nm of the uncoated regions ($0L$), the model works well (Fig. 5a in the main text). These results suggest the existence of another source of NRCs, distinct from the PDs which distribute within the surface region. These defects are highly localized at the surface, therefore, they are most likely the intrinsic surface states (ISSs) generated due to the termination of the crystal lattice.[42] Since all the curves with MoS_2 coating align well with the spatial distribution of PDs without considering ISSs (Figs. S22a–S22c), it suggests that ISSs at III-nitride surfaces are effectively passivated by MoS_2 coating.

Table 4: Summary of the fits shown in Fig. S22 and Fig. 5a (in the main text) using Eq. 8. The data are labeled by the number of MoS_2 MLs (nL) in the corresponding regions. The symbol "*" indicates that the outlier, the point at $d = 0$ nm for regions without MoS_2 ($0L$), is excluded from the fit.

Figure	data	A' (arb. units)	A (arb. units)	L_{eff} (nm)
Fig. S22a	$1L$	0.98 ± 0.10	3.3 ± 1.2	2.4 ± 0.6
Fig. S22b	$2L$	0.96 ± 0.11	3.0 ± 0.8	3.3 ± 0.8
Fig. S22c	$3L$	0.96 ± 0.18	2.8 ± 0.7	3.6 ± 1.3
Fig. S22d	$0L$	1.2 ± 0.2	15 ± 5	0.45 ± 0.59
Fig. S22d	$0L^*$	1, fixed	2.7 ± 1.2	2.5 ± 1.9
Fig. 5a	$0-3L^*$	0.98 ± 0.06	2.9 ± 1.5	3.1 ± 2.0

References

(1) Feltin, E.; Simeonov, D.; Carlin, J.-F.; Butté, R.; Grandjean, N. Narrow UV emission from homogeneous GaN/AlGaN quantum wells. *Appl. Phys. Lett.* **2007**, *90*, 021905.

(2) Novoselov, K. S.; Geim, A. K.; Morozov, S. V.; Jiang, D.; Zhang, Y.; Dubonos, S. V.;

Grigorieva, I. V.; Firsov, A. A. Electric field effect in atomically thin carbon films. *Science* **2004**, *306*, 666–669.

(3) Wang, L.; Meric, I.; Huang, P. Y.; Gao, Q.; Gao, Y.; Tran, H.; Taniguchi, T.; Watanabe, K.; Campos, L. M.; Muller, D. A.; Guo, J.; Kim, P.; Hone, J.; Shepard, K. L.; Dean, C. R. One-dimensional electrical contact to a two-dimensional material. *Science* **2013**, *342*, 614–617.

(4) Castellanos-Gomez, A.; Duan, X.; Fei, Z.; Gutierrez, H. R.; Huang, Y.; Huang, X.; Quereda, J.; Qian, Q.; Sutter, E.; Sutter, P. Van der Waals heterostructures. *Nat. Rev. Methods Primers* **2022**, *2*, 58.

(5) Drouin, D.; Couture, A. R.; Joly, D.; Tastet, X.; Aimez, V.; Gauvin, R. CASINO V2.42 — A fast and easy-to-use modeling tool for scanning electron microscopy and microanalysis users. *Scanning* **2007**, *29*, 92–101.

(6) Evoy, S.; Harnett, C. K.; Keller, S.; Mishra, U. K.; DenBaars, S. P.; Craighead, H. G. Scanning tunneling microscope-induced luminescence studies of defects in GaN layers and heterostructures. *MRS Online Proceedings Library* **1999**, *588*, 19.

(7) Gonzalez, J. C.; Bunker, K. L.; Russell, P. E. Minority-carrier diffusion length in a GaN-based light-emitting diode. *Appl. Phys. Lett.* **2001**, *79*, 1567–1569.

(8) Jahn, U.; Kaganer, V. M.; Sabelfeld, K. K.; Kireeva, A. E.; Lähnemann, J.; Pfüller, C.; Flissikowski, T.; Chèze, C.; Biermann, K.; Calarco, R.; Brandt, O. Carrier diffusion in GaN: A cathodoluminescence study. I. Temperature-dependent generation volume. *Phys. Rev. Appl.* **2022**, *17*, 024017.

(9) Guthrey, H.; Moseley, J. A review and perspective on cathodoluminescence analysis of halide perovskites. *Adv. Energy Mater.* **2020**, *10*, 1903840.

(10) Brunner, D.; Angerer, H.; Bustarret, E.; Freudenberg, F.; Höpler, R.; Dimitrov, R.; Ambacher, O.; Stutzmann, M. Optical constants of epitaxial AlGaN films and their temperature dependence. *J. Appl. Phys.* **1997**, *82*, 5090–5096.

(11) Liu, W. *Ultrafast carrier dynamics in III-nitride nanostructures and LED quantum efficiency*; Ph.D. Dissertation, École Polytechnique Fédérale de Lausanne: Lausanne, Switzerland, 2019.

(12) Negri, M.; Francaviglia, L.; Dumcenco, D.; Bosi, M.; Kaplan, D.; Swaminathan, V.; Salviati, G.; Kis, A.; Fabbri, F.; Fontcuberta i Morral, A. Quantitative nanoscale absorption mapping: A novel technique to probe optical absorption of two-dimensional materials. *Nano Lett.* **2020**, *20*, 567–576.

(13) Chang, Y.-L.; Tan, I.-H.; Zhang, Y.-H.; Bimberg, D.; Merz, J.; Hu, E. Reduced quantum efficiency of a near-surface quantum well. *J. Appl. Phys.* **1993**, *74*, 5144–5148.

(14) Birner, S.; Zibold, T.; Andlauer, T.; Kubis, T.; Sabathil, M.; Trellakis, A.; Vogl, P. nextnano: General purpose 3-D simulations. *IEEE Trans. Electron Devices* **2007**, *54*, 2137–2142.

(15) Triviño, N. V.; Butté, R.; Carlin, J.-F.; Grandjean, N. Continuous wave blue lasing in III-nitride nanobeam cavity on silicon. *Nano Lett.* **2015**, *15*, 1259–1263.

(16) Rossbach, G.; Levrat, J.; Jacopin, G.; Shahmohammadi, M.; Carlin, J.-F.; Ganière, J.-D.; Butté, R.; Deveaud, B.; Grandjean, N. High-temperature Mott transition in wide-band-gap semiconductor quantum wells. *Phys. Rev. B* **2014**, *90*, 201308(R).

(17) Benameur, M. M.; Radisavljevic, B.; Héron, J. S.; Sahoo, S.; Berger, H.; Kis, A. Visibility of dichalcogenide nanolayers. *Nanotechnology* **2011**, *22*, 125706.

(18) Lee, C.; Yan, H.; Brus, L. E.; Heinz, T. F.; Hone, J.; Ryu, S. Anomalous lattice vibrations of single- and few-layer MoS_2. *ACS Nano* **2010**, *4*, 2695–2700.

(19) Nemes-Incze, P.; Osváth, Z.; Kamarás, K.; Biró, L. P. Anomalies in thickness measurements of graphene and few layer graphite crystals by tapping mode atomic force microscopy. *Carbon* **2008**, *46*, 1435–1442.

(20) Li, H.; Zhang, Q.; Yap, C. C. R.; Tay, B. K.; Edwin, T. H. T.; Olivier, A.; Baillargeat, D. From bulk to monolayer MoS_2: Evolution of Raman scattering. *Adv. Funct. Mater.* **2012**, *22*, 1385–1390.

(21) Buscema, M.; Steele, G. A.; van der Zant, H. S. J.; Castellanos-Gomez, A. The effect of the substrate on the Raman and photoluminescence emission of single-layer MoS_2. *Nano Res.* **2014**, *7*, 561–571.

(22) Li, H.; Wu, J.; Huang, X.; Lu, G.; Yang, J.; Lu, X.; Xiong, Q.; Zhang, H. Rapid and reliable thickness identification of two-dimensional nanosheets using optical microscopy. *ACS Nano* **2013**, *7*, 10344–10353.

(23) Ma, B.; Wang, P.; Ren, S.; Jia, C.; Guo, X. Versatile optical determination of two-dimensional atomic crystal layers. *Carbon* **2016**, *109*, 384–389.

(24) de la Peña, F. et al. hyperspy/hyperspy: Release v1.7.5. 2023.

(25) Lähnemann, J.; Flissikowski, T.; Wölz, M.; Geelhaar, L.; Grahn, H. T.; Brandt, O.; Jahn, U. Quenching of the luminescence intensity of GaN nanowires under electron beam exposure: Impact of C adsorption on the exciton lifetime. *Nanotechnology* **2016**, *27*, 455706.

(26) Reshchikov, M. A.; Shahedipour, F.; Korotkov, R. Y.; Wessels, B. W.; Ulmer, M. P. Photoluminescence band near 2.9 eV in undoped GaN epitaxial layers. *J. Appl. Phys.* **2000**, *87*, 3351–3354.

(27) Haigh, S. J.; Gholinia, A.; Jalil, R.; Romani, S.; Britnell, L.; Elias, D. C.; Novoselov, K. S.; Ponomarenko, L. A.; Geim, A. K.; Gorbachev, R. Cross-sectional

imaging of individual layers and buried interfaces of graphene-based heterostructures and superlattices. *Nat. Mater.* **2012**, *11*, 764–767.

(28) Dumcenco, D.; Ovchinnikov, D.; Marinov, K.; Lazić, P.; Gibertini, M.; Marzari, N.; Sanchez, O. L.; Kung, Y.-C.; Krasnozhon, D.; Chen, M.-W.; Bertolazzi, S.; Gillet, P.; Fontcuberta i Morral, A.; Radenovic, A.; Kis, A. Large-area epitaxial monolayer MoS_2. *ACS Nano* **2015**, *9*, 4611–4620.

(29) Castellanos-Gomez, A.; Quereda, J.; van der Meulen, H. P.; Agraït, N.; Rubio-Bollinger, G. Spatially resolved optical absorption spectroscopy of single- and few-layer MoS_2 by hyperspectral imaging. *Nanotechnology* **2016**, *27*, 115705.

(30) Zhang, Z.; Qian, Q.; Li, B.; Chen, K. J. Interface engineering of monolayer MoS_2/GaN hybrid heterostructure: Modified band alignment for photocatalytic water splitting application by nitridation treatment. *ACS Appl. Mater. Interfaces* **2018**, *10*, 17419–17426.

(31) Jain, S. K.; Kumar, R. R.; Aggarwal, N.; Vashishtha, P.; Goswami, L.; Kuriakose, S.; Pandey, A.; Bhaskaran, M.; Walia, S.; Gupta, G. Current transport and band alignment study of MoS_2/GaN and MoS_2/AlGaN heterointerfaces for broadband photodetection application. *ACS Appl. Electron. Mater.* **2020**, *2*, 710–718.

(32) Ermolaev, G. A.; Stebunov, Y. V.; Vyshnevyy, A. A.; Tatarkin, D. E.; Yakubovsky, D. I.; Novikov, S. M.; Baranov, D. G.; Shegai, T.; Nikitin, A. Y.; Arsenin, A. V.; Volkov, V. S. Broadband optical properties of monolayer and bulk MoS_2. *npj 2D Mater. Appl.* **2020**, *4*, 21.

(33) Barker, A. S.; Ilegems, M. Infrared lattice vibrations and free-electron dispersion in GaN. *Phys. Rev. B* **1973**, *7*, 743–750.

(34) Pastrňák, J.; Roskovcová, L. Refraction index measurements on AlN single crystals. *phys. stat. sol. (b)* **1966**, *14*, K5–K8.

(35) Martin, G.; Botchkarev, A.; Rockett, A.; Morkoç, H. Valence-band discontinuities of wurtzite GaN, AlN, and InN heterojunctions measured by x-ray photoemission spectroscopy. *Appl. Phys. Lett.* **1996**, *68*, 2541–2543.

(36) Rinke, P.; Winkelnkemper, M.; Qteish, A.; Bimberg, D.; Neugebauer, J.; Scheffler, M. Consistent set of band parameters for the group-III nitrides AlN, GaN, and InN. *Phys. Rev. B* **2008**, *77*, 075202.

(37) Grandjean, N.; Damilano, B.; Massies, J.; Neu, G.; Teissere, M.; Grzegory, I.; Porowski, S.; Gallart, M.; Lefebvre, P.; Gil, B.; Albrecht, M. Optical properties of GaN epilayers and GaN/AlGaN quantum wells grown by molecular beam epitaxy on GaN(0001) single crystal substrate. *J. Appl. Phys.* **2000**, *88*, 183–187.

(38) Han, D.-P.; Fujiki, R.; Takahashi, R.; Ueshima, Y.; Ueda, S.; Lu, W.; Iwaya, M.; Takeuchi, T.; Kamiyama, S.; Akasaki, I. n-type GaN surface etched green light-emitting diode to reduce non-radiative recombination centers. *Appl. Phys. Lett.* **2021**, *118*, 021102.

(39) Chichibu, S. F.; Uedono, A.; Kojima, K.; Ikeda, H.; Fujito, K.; Takashima, S.; Edo, M.; Ueno, K.; Ishibashi, S. The origins and properties of intrinsic nonradiative recombination centers in wide bandgap GaN and AlGaN. *J. Appl. Phys.* **2018**, *123*, 161413.

(40) Haller, C.; Carlin, J.-F.; Jacopin, G.; Liu, W.; Martin, D.; Butté, R.; Grandjean, N. GaN surface as the source of non-radiative defects in InGaN/GaN quantum wells. *Appl. Phys. Lett.* **2018**, *113*, 111106.

(41) Chen, Y.; Haller, C.; Liu, W.; Karpov, S. Y.; Carlin, J.-F.; Grandjean, N. GaN buffer growth temperature and efficiency of InGaN/GaN quantum wells: The critical role of nitrogen vacancies at the GaN surface. *Appl. Phys. Lett.* **2021**, *118*, 111102.

(42) Shockley, W. On the surface states associated with a periodic potential. *Phys. Rev.* **1939**, *56*, 317–323.
